# Supplementary material for: Intra-horn insemination in the alpaca Vicugna pacos: Copulatory wounding and deep sperm deposition
Source: PLoS One. 2024 Apr 17;19(4):e0295882. doi: 10.1371/journal.pone.0295882 (PMC11023217; doi:10.1371/journal.pone.0295882)
Supplement: S1 Table — Percentage of pixels with blood in each region of the reproductive tract of unmated females, and females mated 1 hr or 24 hrs before photographing the tract. (PDF) [file pone.0295882.s001.pdf]

S1 Table 1. Blood per region. Percentage of pixels with blood in each region of the reproductive tract of unmated females, and females mated 1 hr or 24 hrs before photographing the tract. Higher percentages of blood were seen in the upper reproductive tract of mated females

mating condition	alpaca ID	hymen	vagina	cervix	uterine body	R horn	L horn
**unmated**	A034	0	0	0	0	0	0
**unmated**	A037	0	0	0	0	0	0
**unmated**	A040	0	0.23	0	0.11	0	0
**unmated**	A041	0	0.09	0.23	0.05	0	0
**unmated**	A043	0	0	0	0	0	0
**unmated**	A044	0.07	0	0.06	0	0	0
**mated +1hr**	A024	3.55	0	5.43	1.88	4.17	0.44
**mated +1hr**	A025	11.09	0.61	2.63	3.08	33.87	21.8
**mated +1hr**	A031	0.13	0.09	6.3	11.12	2.7	4.6
**mated +1hr**	A032	1.94	2.54	20.74	16.08	29.5	64
**mated +24hrs**	A030	0.03	0	0.82	4.25	7.18	9.85
**mated +24hrs**	A033	0.19	0	3.85	0.9	0.04	0.08
**mated +24hrs**	A035	5.19	0.02	0.77	2.08	12.62	5.1
**mated +24hrs**	A036	8.43	6.69	0	0	0	0
**mated +24hrs**	A039	0.14	1.51	14	24.87	20.02	16.67

**mated +24hrs**	A042	0.06	0	76.19	60.35	13.51	15.47

.
