# Supplementary material for: Intra-horn insemination in the alpaca Vicugna pacos: Copulatory wounding and deep sperm deposition
Source: PLoS One. 2024 Apr 17;19(4):e0295882. doi: 10.1371/journal.pone.0295882 (PMC11023217; doi:10.1371/journal.pone.0295882)
Supplement: S3 Table — (PDF) [file pone.0295882.s003.pdf]

**Animal**						**Cytology (0-4)**						
	**Age (yr)**	**Breeding History**	**Ovary gross findings**	**internal tract hemorrhage**	**Breeding length (min)**	**vagina**	**cervix**	**uterine body**	**left uterine horn**	**right uterine horn**	**oviducts**	**left oviduct**
**Bred day of sacrifice- 1 hr post breeding**												
**31**	5	3 crias since 2015; last in 2018 (1 year prior)	F10 RO MSF LO		28	1 RBC *Live Dead stain only*						
**32**	17.5	4 crias since 2013; last in 2018 (1 year prior)	2F10 RO MSF LO		12	*Live Dead stain only*						
**24**	9	??	2F10 RO	caudal vagina, cervix, horn tips	13	3 RBC *Live Dead stain only*		2 RBC			1 RBC	
**25**	MB Erica	??			20	*Live Dead stain only*						
**Bred 24 hrs. prior to sacrifice**						s						
**30**	7	bred 1 time in 2019 (year of sacrifice)- not pregnant	10F RO	both uterine papillae		3 RBC, 1 NLE	1 N 1L 1E	3 RBC, 2 N 2L 3E	1 N, 1L, 2E	1 N, 1L, 2E	1 RBC	3L
**33**	9	last cria 2016 (3 years prior)	12F LO MSF RO	slight RH tip, 0 LH tip, black pigment cranial vagina, slight cervix	15	2N (images), 1 MP (image)	2N, 2 RBC, 2L	2E, 2N, 2 RBC, 2L	1E,1RBC, 1L,2mucous strands (images)	1E,1N, 3RBC, 3L		uterine papilla- 0 sperm, 2 E, 2RBC, 2L (images- mixed cells, mixed cell clusters)
**35**	2	uncomfortable when bred by Lester 11/21/19, her first breeding; so stopped and bred 20 mins. to Revolution		hymen and both horn tips; curled up hair in left horn; small uterus: images saved	uncomfortable for first male, second normal for 20 minutes	1N, 1E	1E, 2N, 2L	1E, 1N, 2L	1E, 1, 1L	1E, 1N, 2L		uterine papilla- 1E, 1N, 1L, 1 snowballs oviduct- 3E, 1L

Animal		Sperm conc. (0-4)			
	right oviduct	vagina	cervix	uterine horns	oviducts
**Bred day of sacrifice- 1 hr post breeding**					
**31**		1 (100% live)	3 (100% live)	left 1 (5% motile)<br>right 1 (0% motile)	left 4<br>right 4
**32**		1 (100% live)	3 (100% live)	left 2<br>right 1	left 2 (100% live)<br>right 0, few sperm with head and midpiece defects present
**24**		1 (90% live)	no sample	right 1 (70% motile)<br>left 3	4 left and right (PB slides)
**25**		3 (40% motile)	1 (10% motile)	left 2 (60% motile)<br>right 2 (50% motile)	left 1<br>right 0
**Bred 24 hrs. prior to sacrifice**					
**30**	3L	0	no slide	left1<br>right 0	left 1<br>right 0
**33**	right uterine papilla- 1E, 1N, 1RBC, 1L	0	0	0	0
**35**	uterine papilla- 1E, 1N, 1L<br>oviduct- 2E, 1L, 1 snowballs	1	2	0	left 3 right 1

Animal										
Bred day of sacrifice- 1 hr post breeding										
31										
32										
24										
25										
Bred 24 hrs. prior to sacrifice										
30										
33										
35										

**36**	8	bred in past but never pregnant; turned out with male in herd; intermittent 25-30 min. breeding 12/5/19	PB has image, right ovarian mass probably a 13.0CL; 9.2FLO		25-30 interrupted	1E (squamous and columnar) pH 8.0,	1E, 1RBC	1E, 3RBC pH 7.6	1E, 2RBC	1E, 2RBC		1E, 3RBC, low cellularity both oviducts
**39**	10.5	5 cias; last in 2019 (1 year prior)	LO lost FRO?	RH>LH at tips, slight at vulva, greater at cervix	15	1 SQE, 1 col E, 4RBC, 3N, 2L	1 col E, 3RBBC, 4N, 2L ; mucus= 4 degen E, 1 E, 1 RBC, 4N, 1L, 1 MP	4 Col E, 3RBC, 4N, 1L, sheets of col E	4 Col E, 3RBC, 4N, 2L, sheets of Col E	1 Col E, 4RBC, 1N, 1L		lost
**42**	13	3 crias, last in 2019 (1 year prior)	8.4FRO, MSF LO	extensive hemorrhage cerix and both horns, none in vagina	25 mins @ 20 hrs, PTS; SB has WG stained slides	pH 7, 1 E few, 1 N few, 1L few, poor slide	pH 7, 1 sperm rare, 1 degen E, 1RBC, 4N, 1L, clusters of small E cells	pH 7, 1 sperm, 2 col E, 2RBC, 3N, 1L	pH 8, 0 sperm, 1E rare, 4RBC, 1N, 1L, most areas of slide ~ blood	pH 8, 0 Sperm, 2E, 4RBC, blood smear, 1N, 1L, some clusters Cub E, some E with small nuc.		proximal: 1 sperm, prim. small nuc E, 2RBC, 1N rare, 1L; distal-mid: 0 sperm, 3E small nuc cluster and solitary, 1RBC, 0N, 1L, snake eyes image
**Not Bred prior to sacrifice**												
**34**	7	never bred always NR	10.2F RO 7.0 Pearl (CL?) LO	relaxed cervix, fluid in uterine lumen (SG 1023, TP 2.8)		1E, image of squamous E cells	2E	2E, sheets of E, low cellularity, 1L	2E, 1L	2E, 1L, 1 RBC	image of E and white circles	utertine papilla- 3E, 1L, 2 RBC, oviduct- 1E, 2RBC, 1LC
**37**	3	never exposed to a male	8F LO, MSF RO	some fluid in uterus PB has images	never exposed to a male	unrecognizable pale cells?? pH 8.0	1E, 1RBC image of sheets of epith cells at 1000X	2E, pH 8.0	many damaged, unrecog, cells	many damaged, unrecog, cells	right and left uterine papillae bad slides	1E, 3RBC
**Pregnant at sacrifice**												
**038 (approx. 3 mos pregnant by history and size of fetus)**	12	3 previous crias prior to sacrifice which revealed pregnancy (turned out with male in herd 6 mos, prior to sacrifice)	1 CL on each ovary	video and images of entire tract and pregnancy; saved fetus and placenta, 2 CLs but only one pregnancy	pasture turnout starting June 2019	1E pH 8.0	2 RBC	1E, 2 RBC, images pH 8.0	3RBC	1E, 2 RBC		uterine palillae- 2RBC oviduct- 1E, 1-2 RBC, sheets of cells with oval nuclei, some without cytoplasm
**Receptive and not bred**												

**36**	2 RBC	0	0	0	left 0 right 1							
**39**	4 Col E, 1 RBC, snowball image	0	0	0	0 right only							
**42**	middle: 1 sperm rare, 4 E small nuc clusters and solitary, 4RBC, 1 N rare, 1L	0	1 rare	left and right 0	1 L prox, 1 R middle rare							
**Not Bred prior to sacrifice**												
**34**	uterine papilla- 1E. 1L oviduct- 2E, 1RBC, 2L											
**37**	1E, 3RBC, 1 unidentifiable cells											
**Pregnant at sacrifice**												
**038 (approx. 3 mos pregnant by history and size of fetus)**	uterine papilla- 1E, 2RBC oviduct- 1E, 1RBC											
**Receptive and not bred**												

36										
39										
42										
Not Bred prior to sacrifice										
34										
37										
Pregnant at sacrifice										
038 (approx. 3 mos pregnant by history and size of fetus)										
Receptive and not bred										

**40**	13	8 crias, last in 2013 (7 years prior)		NA	SB has WG stained slides	1E cuboidal; mostly degen. cells, poor slide	4E cub > short col, 4 RBC, 1N	4E, 4RBC, 1N, 1L	4E- small nuceli in clusters, 4RBC, 1 N rare, 1L ; some E elongated and some normal nucelus	4E- same variations as LH, 4RBC, 1N, 1L		4E- sheets and clusters ans snowballs; pteinaceous material in backgraound in some areas; 1RBC, 1 N rare
**41**	14	3 crias, last in 2019 (1 year prior)	large FLO MSF RO	2 small old hemorrhages each horn; watery fuid both horns and vagina	SB has WG stained slides	3 E- cuboidal and clusters, 3RBC, 1N rare, 1 L, nucleus shape of E varies	4E- cuboidal clusters, 2RBC, 1N rare, 1L rare, snowballs, some E large nuclei	3 E clusters, mostly cuboidal, 4RBC		4E- cuboidal clusters with small nuclei, 4RBC, 1L		2E cub and sheets
**43**	10	4 crias; last in 2019 (1 year prior)	10.5FRO MSF LO	NA	very receptive 6/23 SB has WG stained slides	pH not measured, 1 col E rare, 0 RBC, 4N, 2L	pH 7, 4E v var. size nuc., 2 RBC, E in sheets, clusters, and solitary, some mucus strands; thick mucus collected: 4 N, pus, many degen. N	pH 7, 3E small cub. and col. ,1RBC, some E clusters, some single	pH 7, 4E clusters and col., 1 RBC, 1 N rare, var. size nuc. E	pH 7, 3 E var size nuc., 2 RBC, 1 N, 1 L, some areas blood		mid- 3 E cub, small nuc., small cluters, small sheets, 2 RBC
**44**	11	2 crias, last in 2018 (2 years prior)	9.3FLO MSF RO	NA	mildly receptive 6/23 SB has WG stained slides	pH 7, 1 Sq E, 1 N rare	pH 7, 4 E cub, single, sheets, clusters	pH 7, 3E, prim cub., clsters, and singles, 2 RBC, 1 N rare,	pH 7, 4E cub. clusters and singles., 3 RBC, 1 N rare	pH 7, 4E prim. cub., sheets, singles, clusters, var. nuc size E, some areas many RBCs		prox- 4E prim. cub, clusters, sheets, singles, 3 RBC 1 N rare
				LEGEND: cytology- RBC = red blood cell; E= epithelial cell; N= neutrophil; L= lymphocyte; blank space= no data available; snowballs= white circular objects of varying size- identity unknown so far	1 = 1-25%<br>2 = 26-50%<br>3 = 51-75%<br>4 = 76-100%<br>of fields on entire slide<br>slides made by impression smear strained with Diff Quik or Dip Quick<br>MP = macrophage							

**40**	4E- sheets and clusters, snowballs, !RBC, 1N rare											
**41**	3E cub, elong. nuclei, columnar, 2RBC											
**43**	same as left											
**44**	prox- 4 E, prim cub., clusters, sheets, singles, 2 RBC, 1 N rare											

40										
41										
43										
44										
