# Supplementary figures and images for: Intra-horn insemination in the alpaca Vicugna pacos: Copulatory wounding and deep sperm deposition
Source: PLoS One. 2024 Apr 17;19(4):e0295882. doi: 10.1371/journal.pone.0295882 (PMC11023217; doi:10.1371/journal.pone.0295882)

ALP024

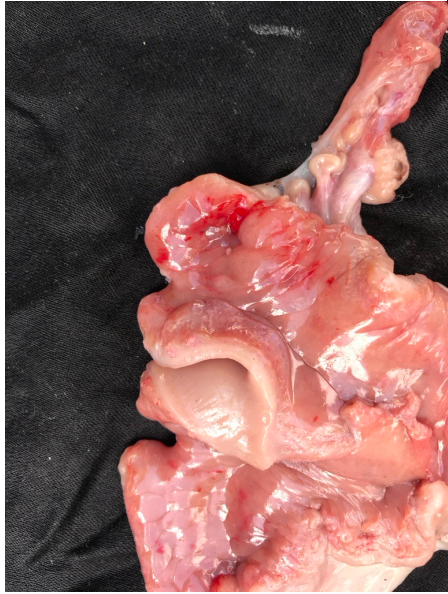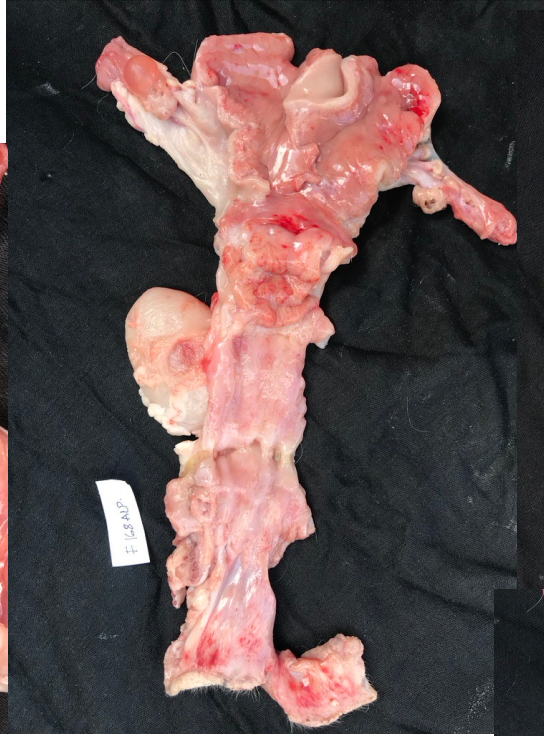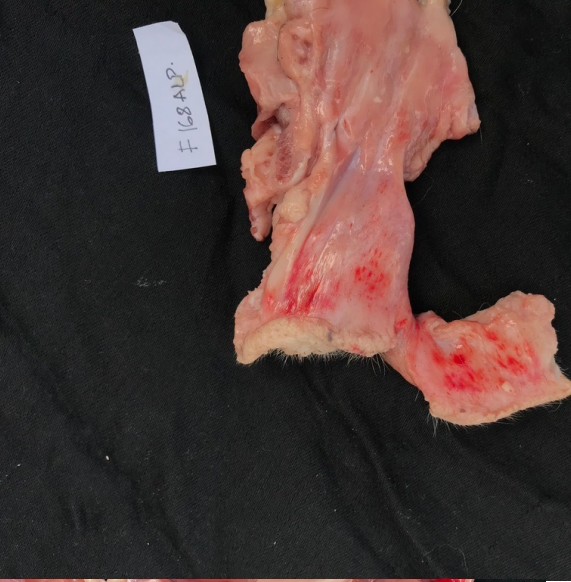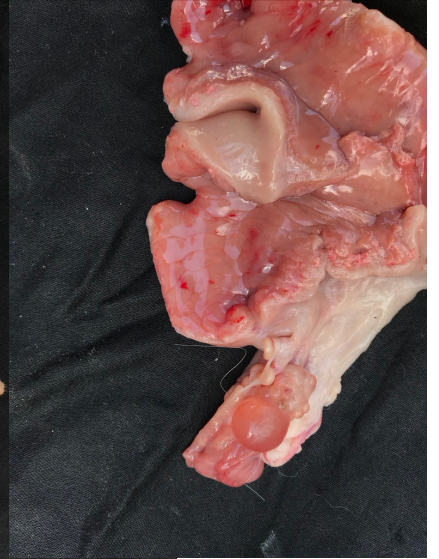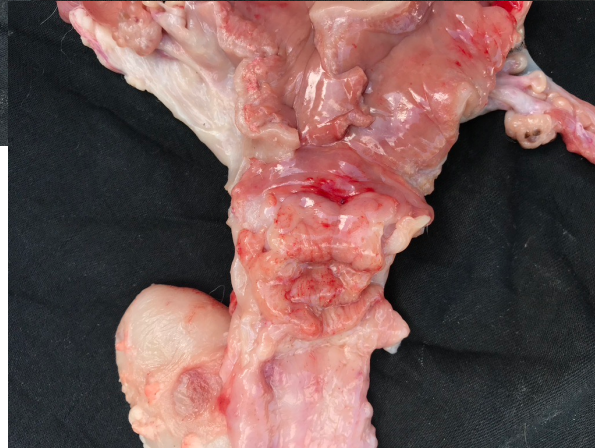

ALPF025

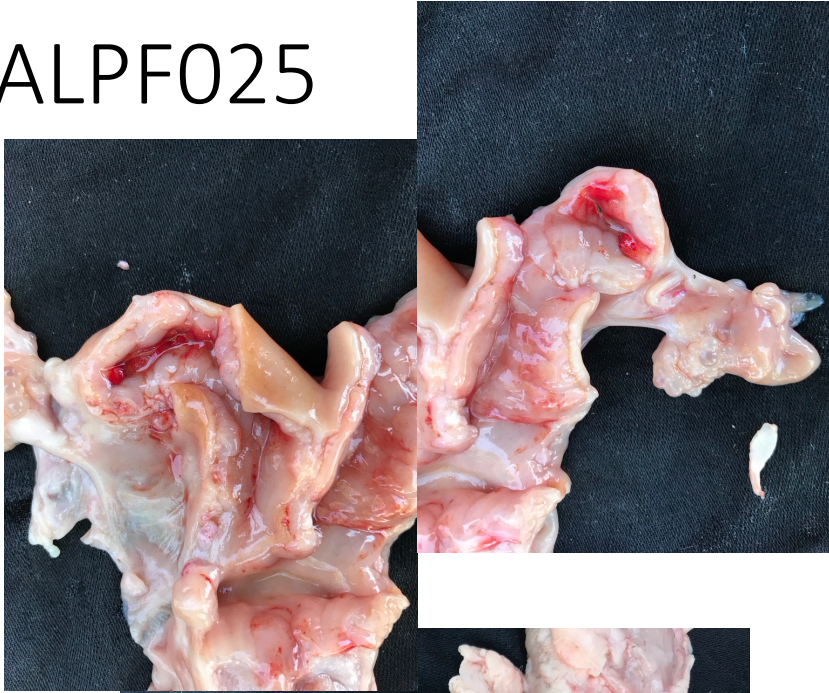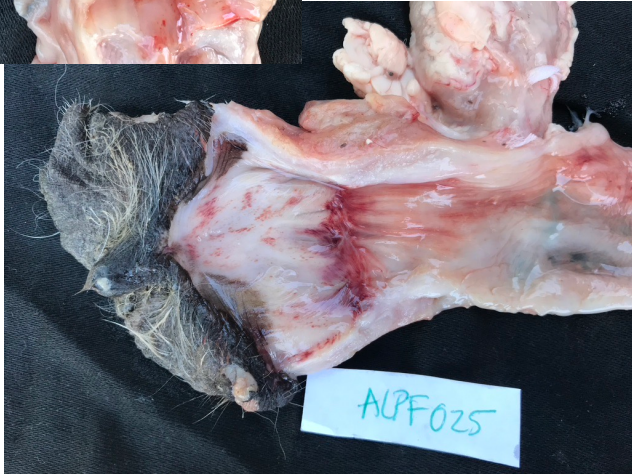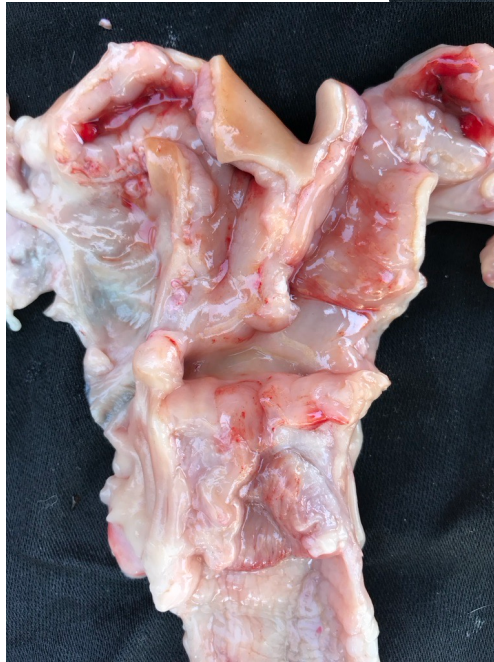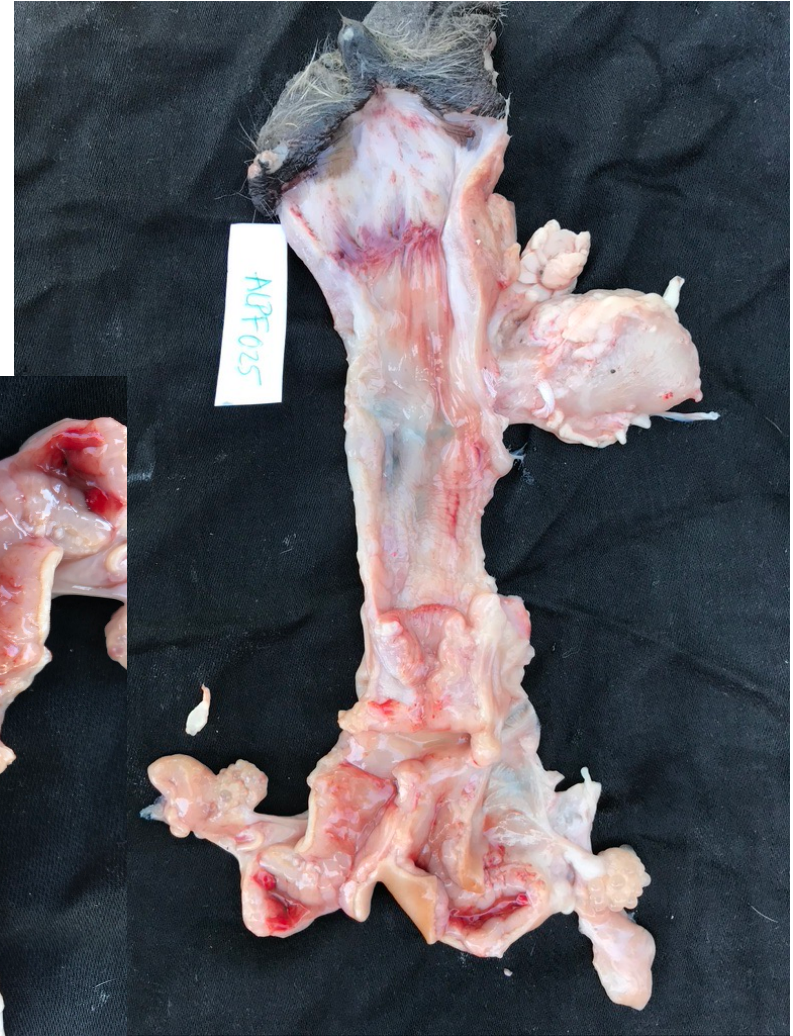

ALPF030

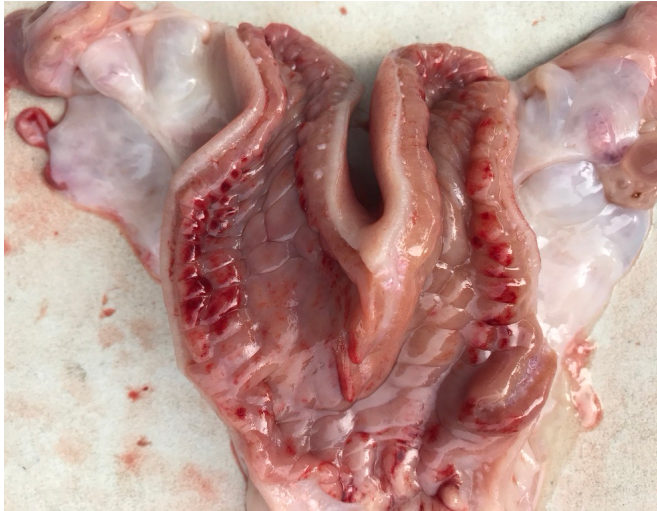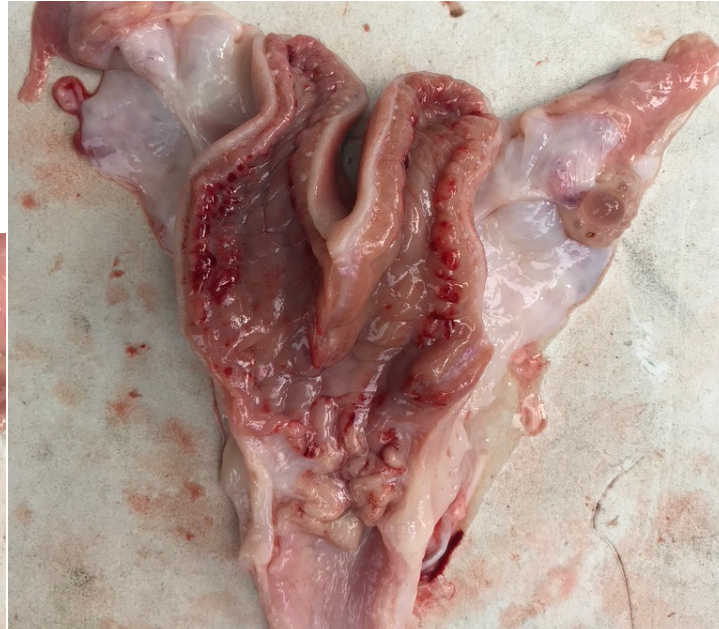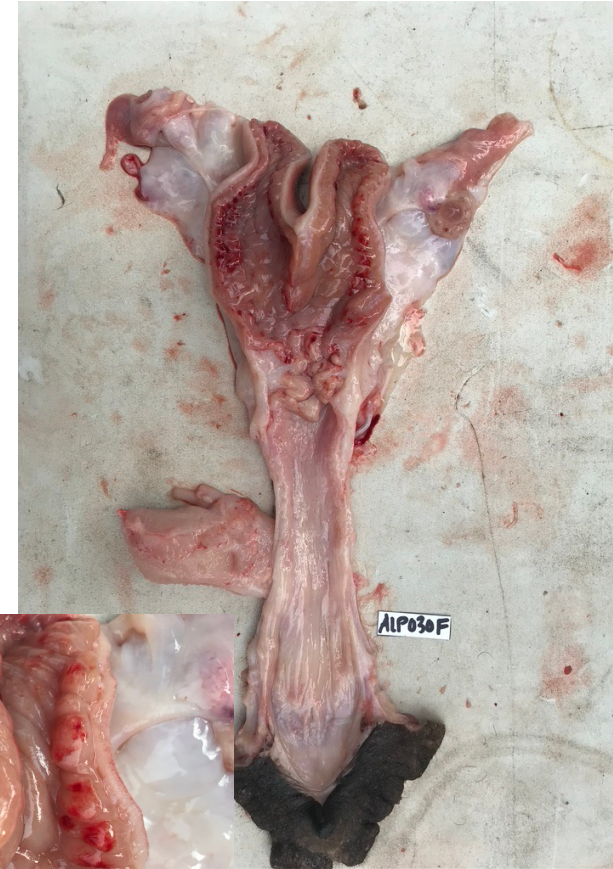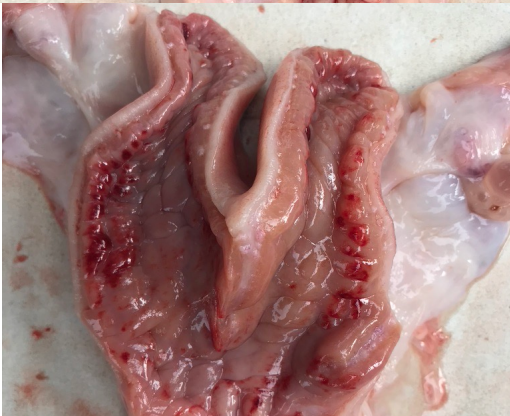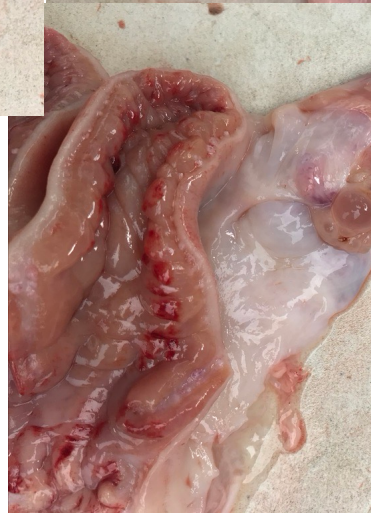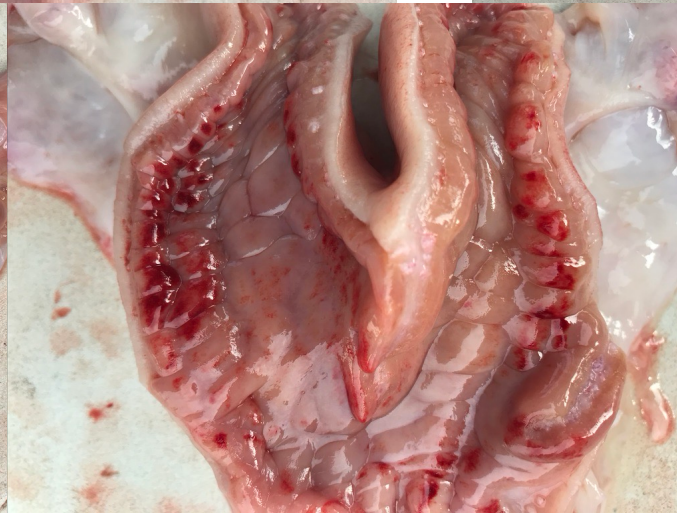

ALPf031

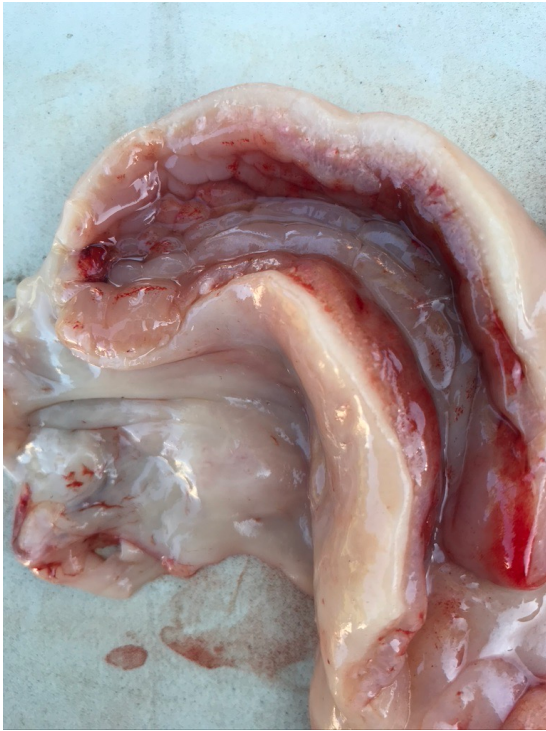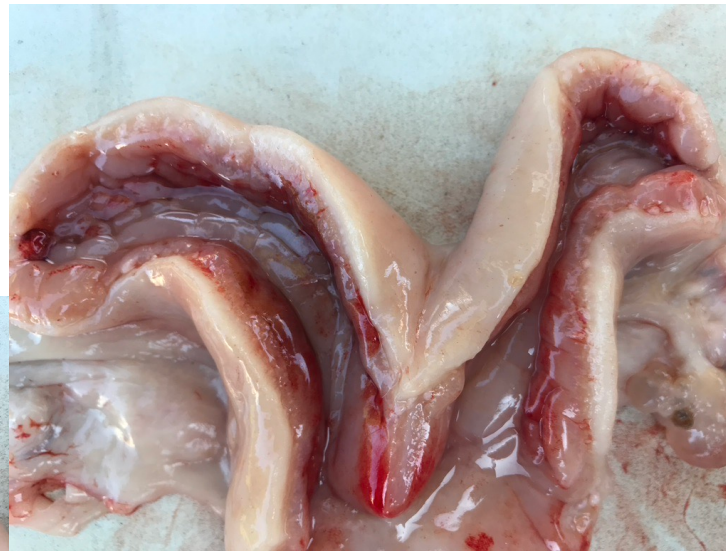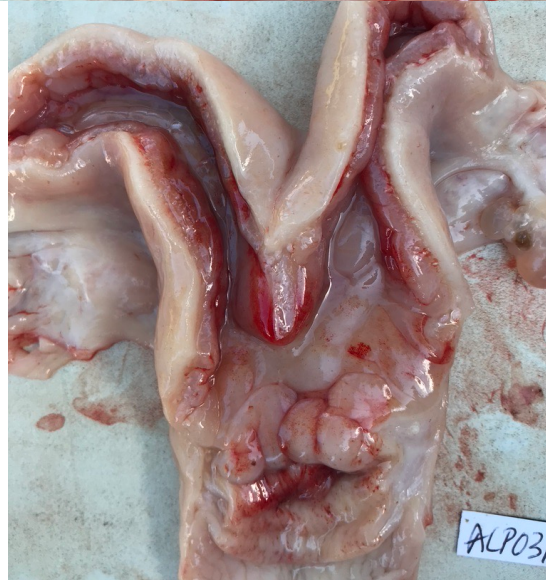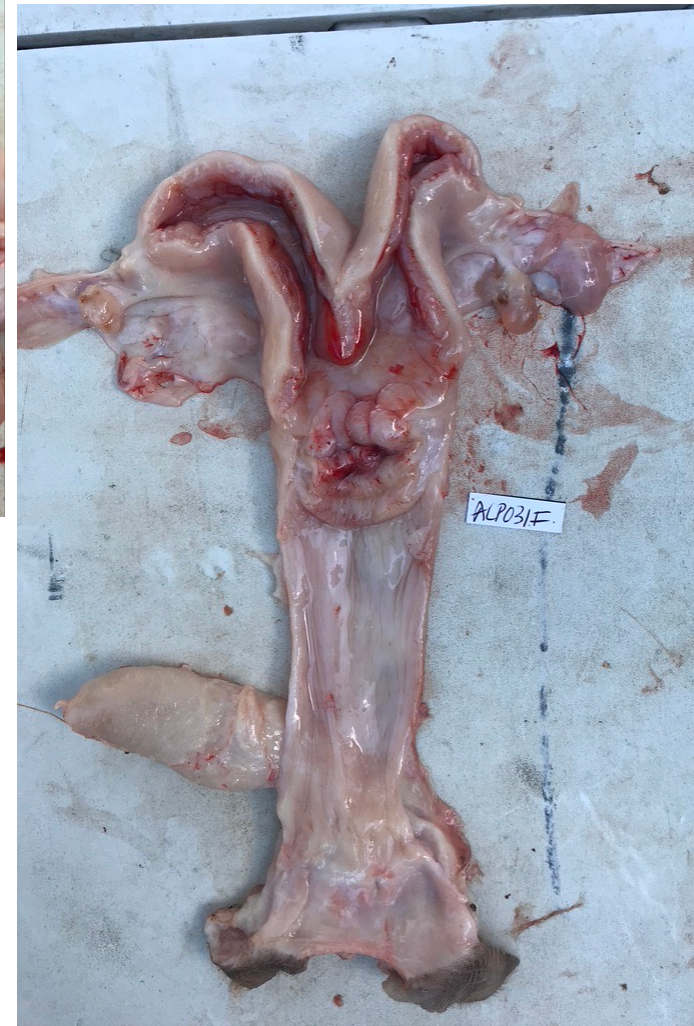

ALPF032

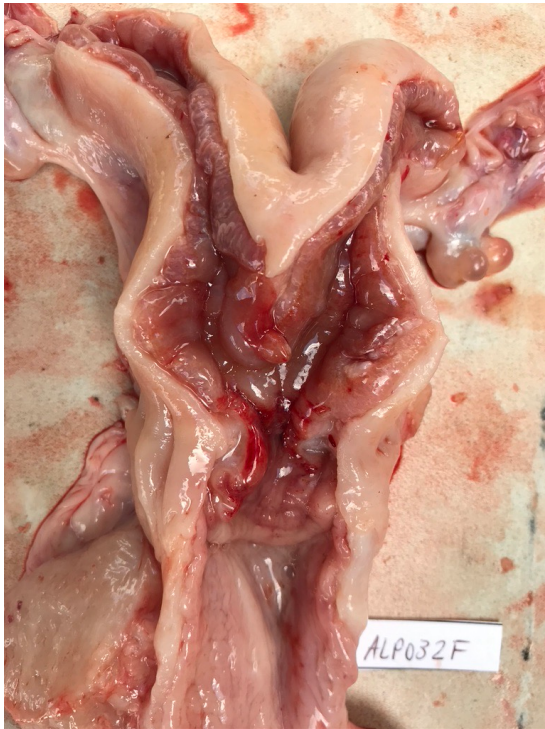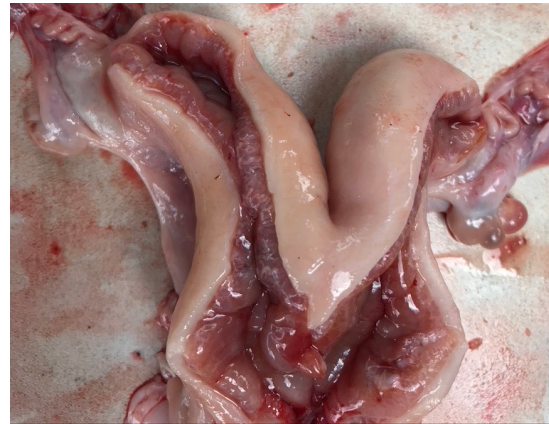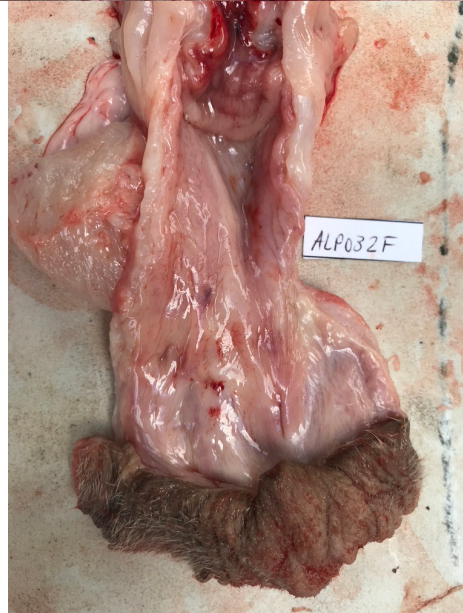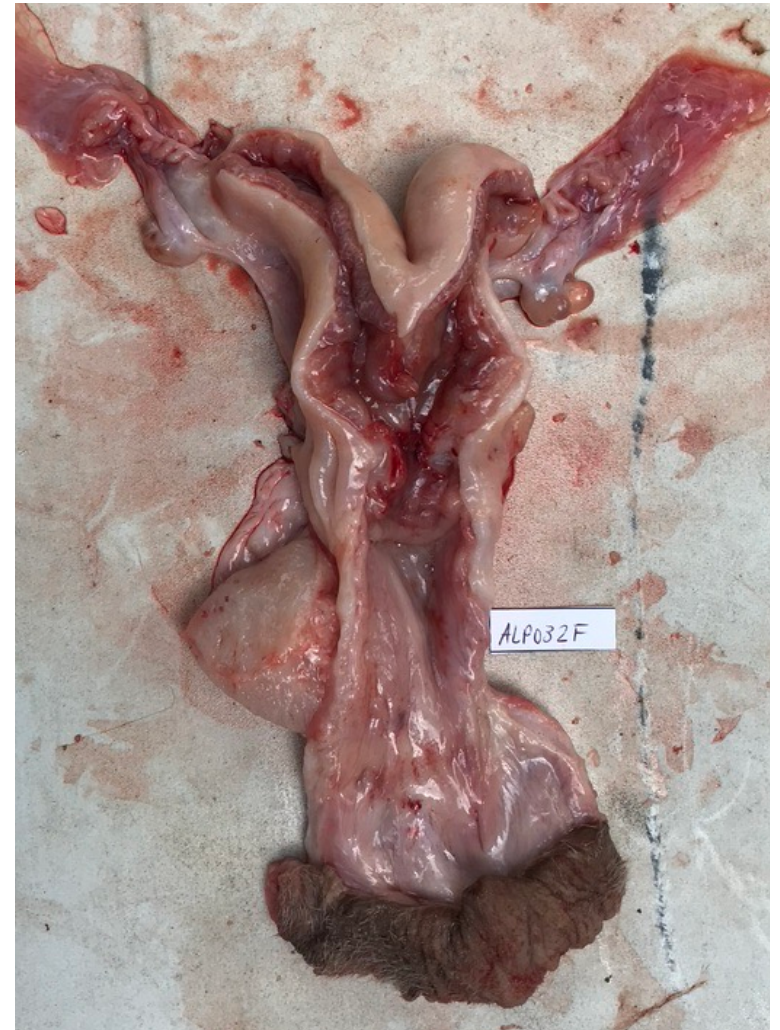

ALPF033

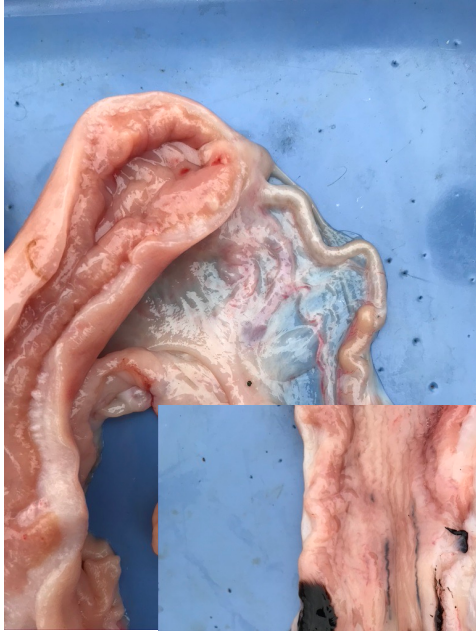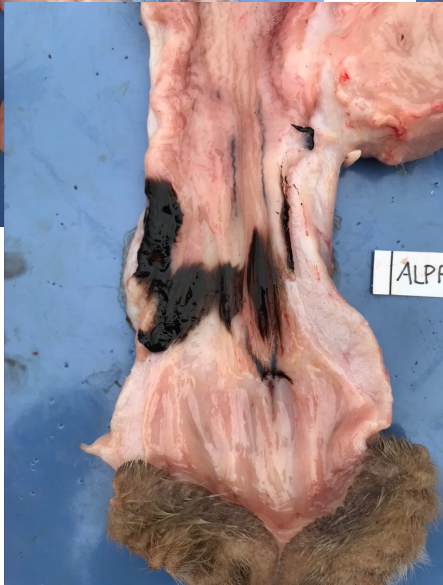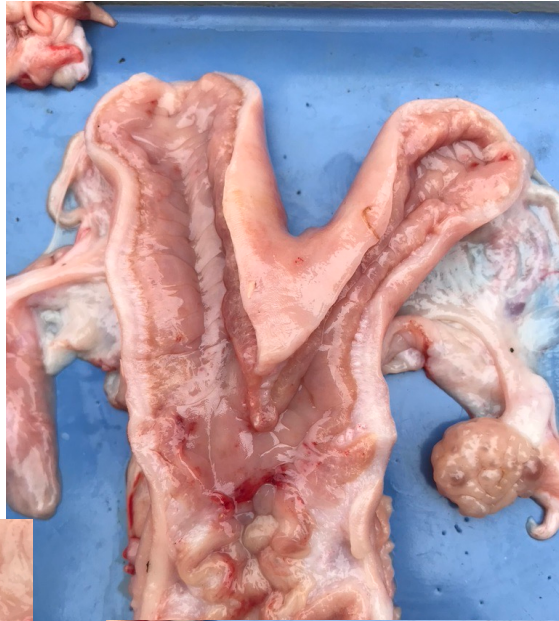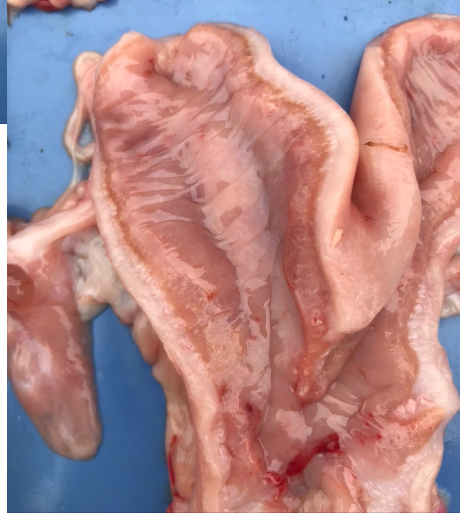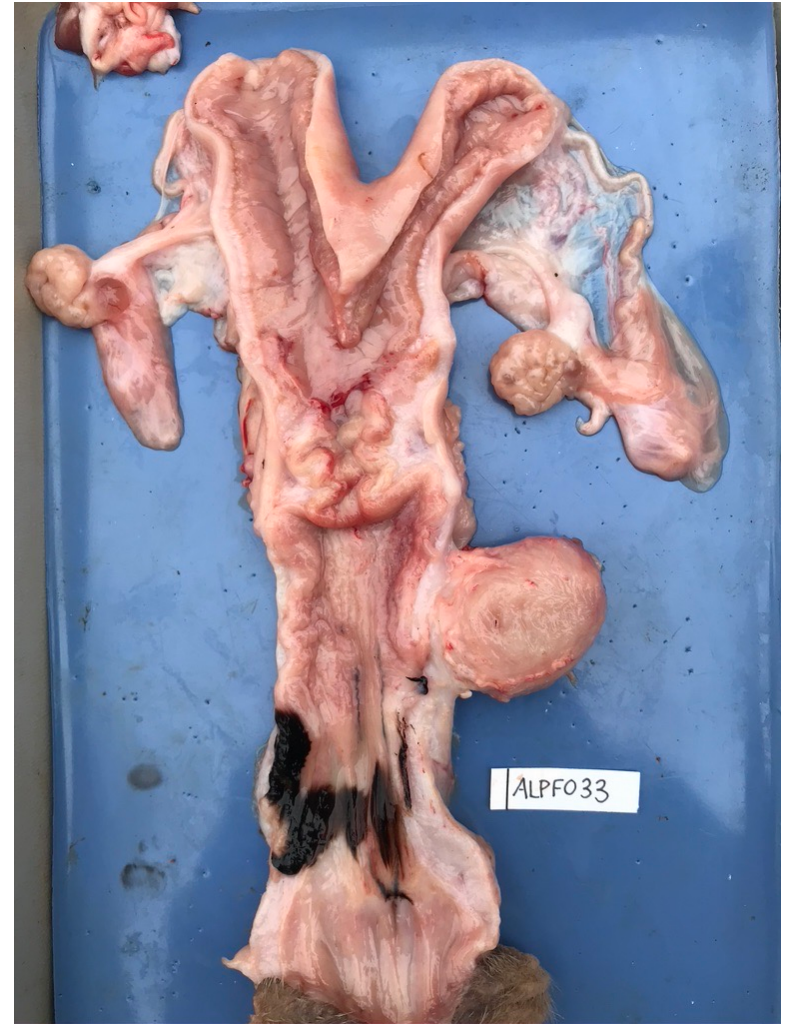

ALPF035

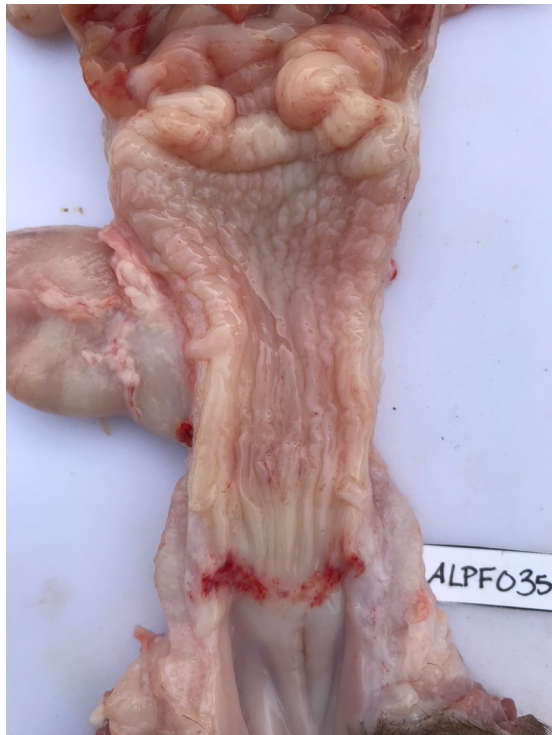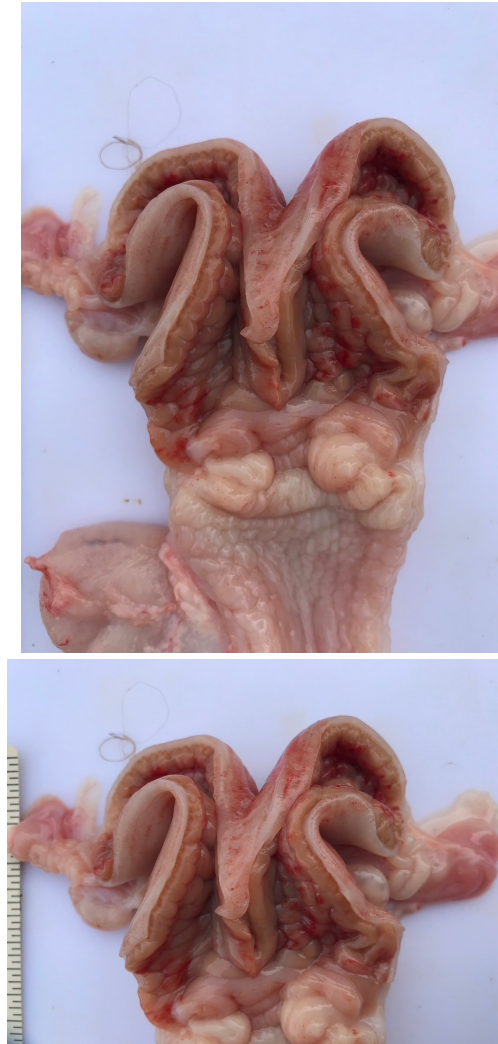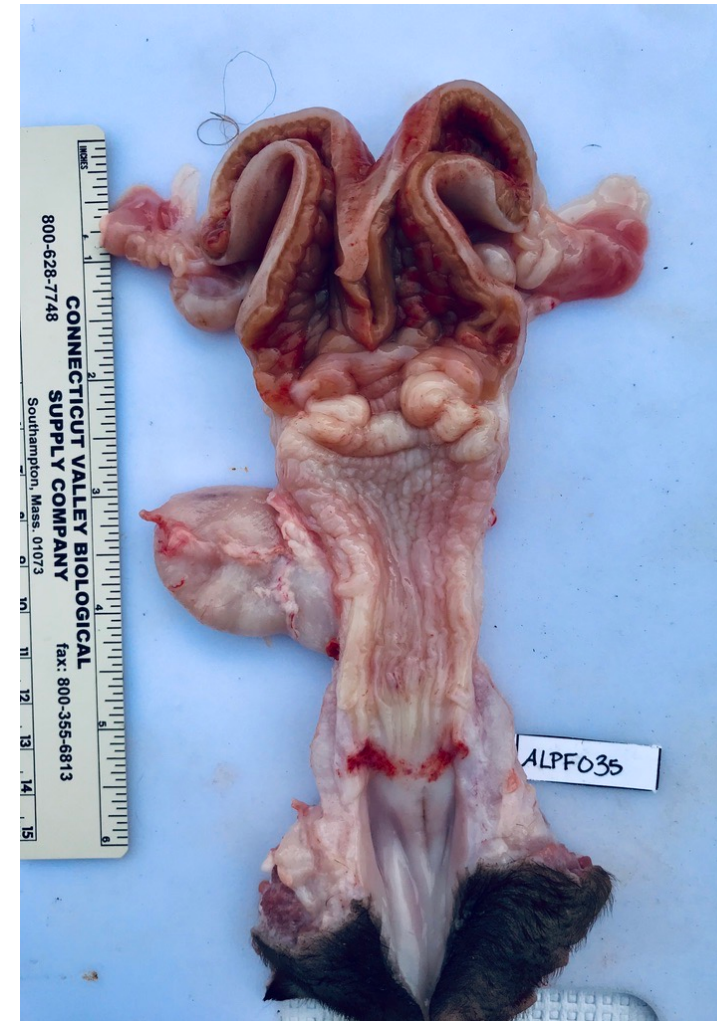

ALPF034

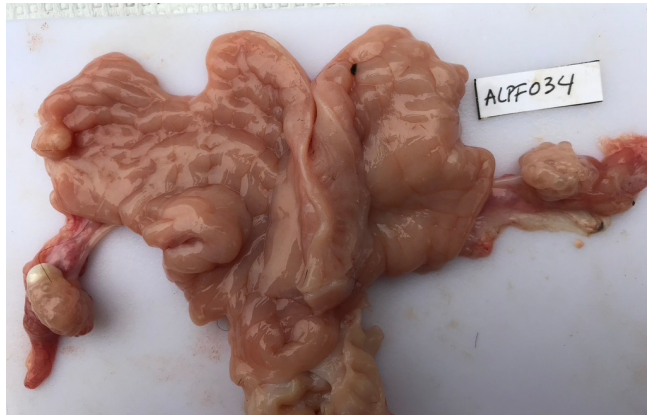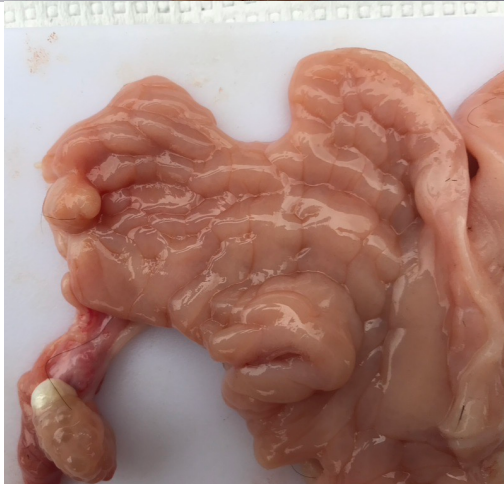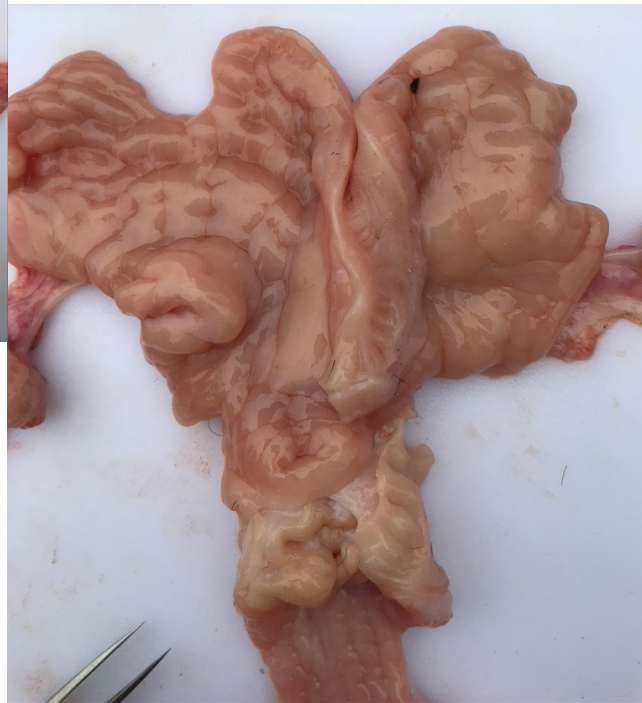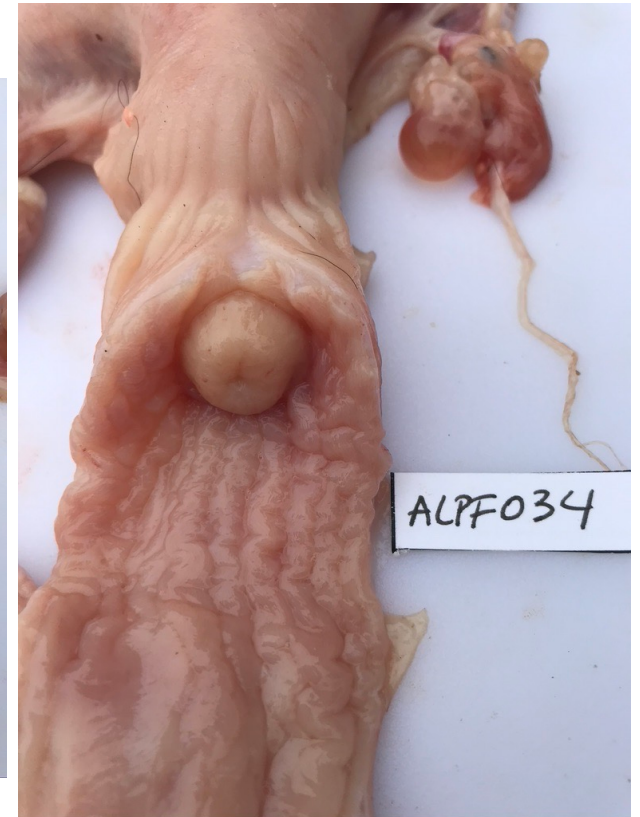

ALPF036

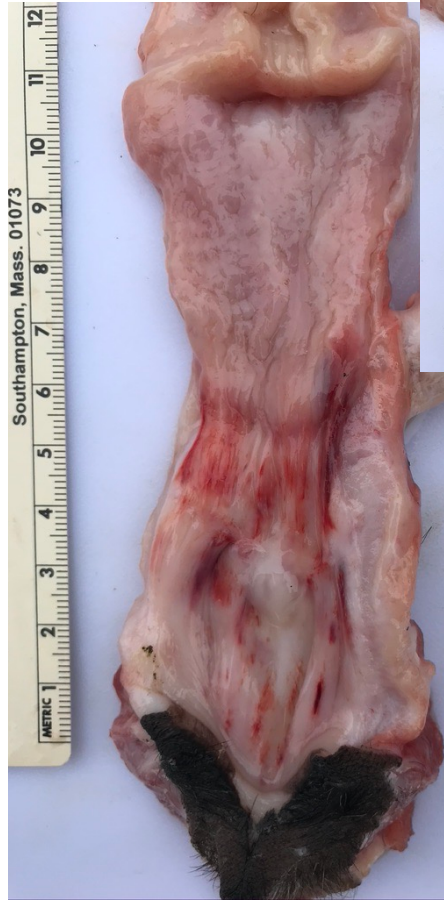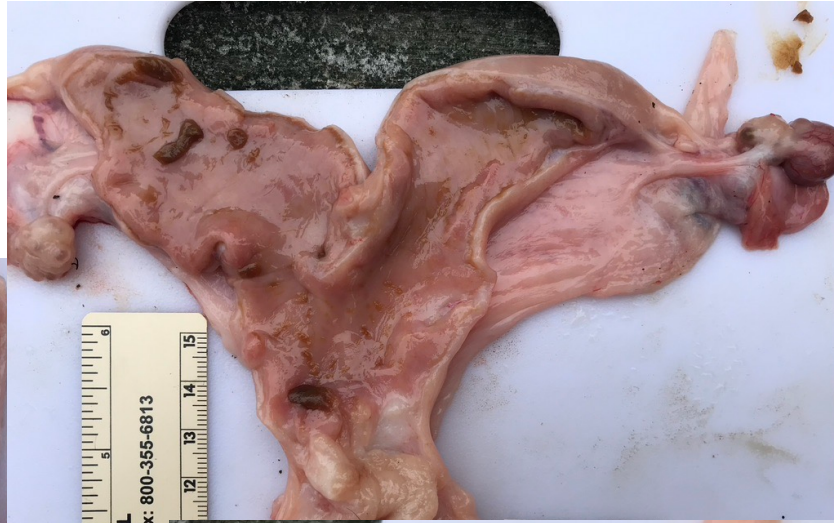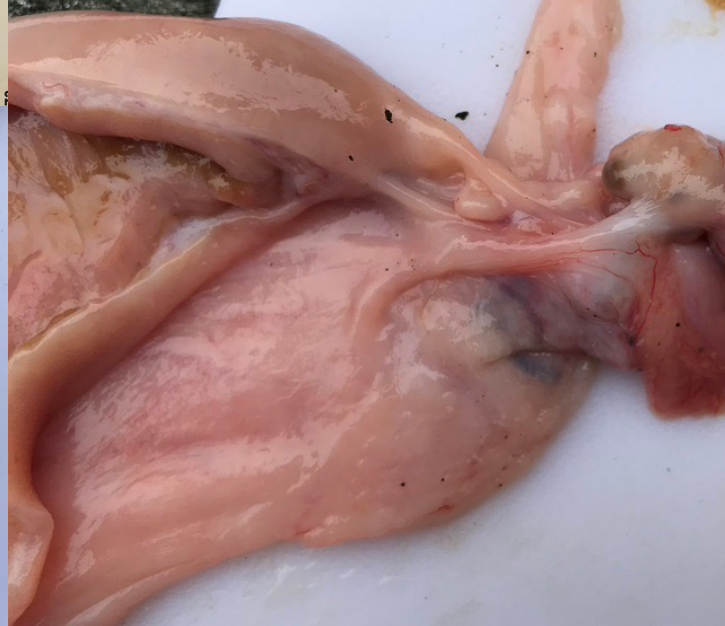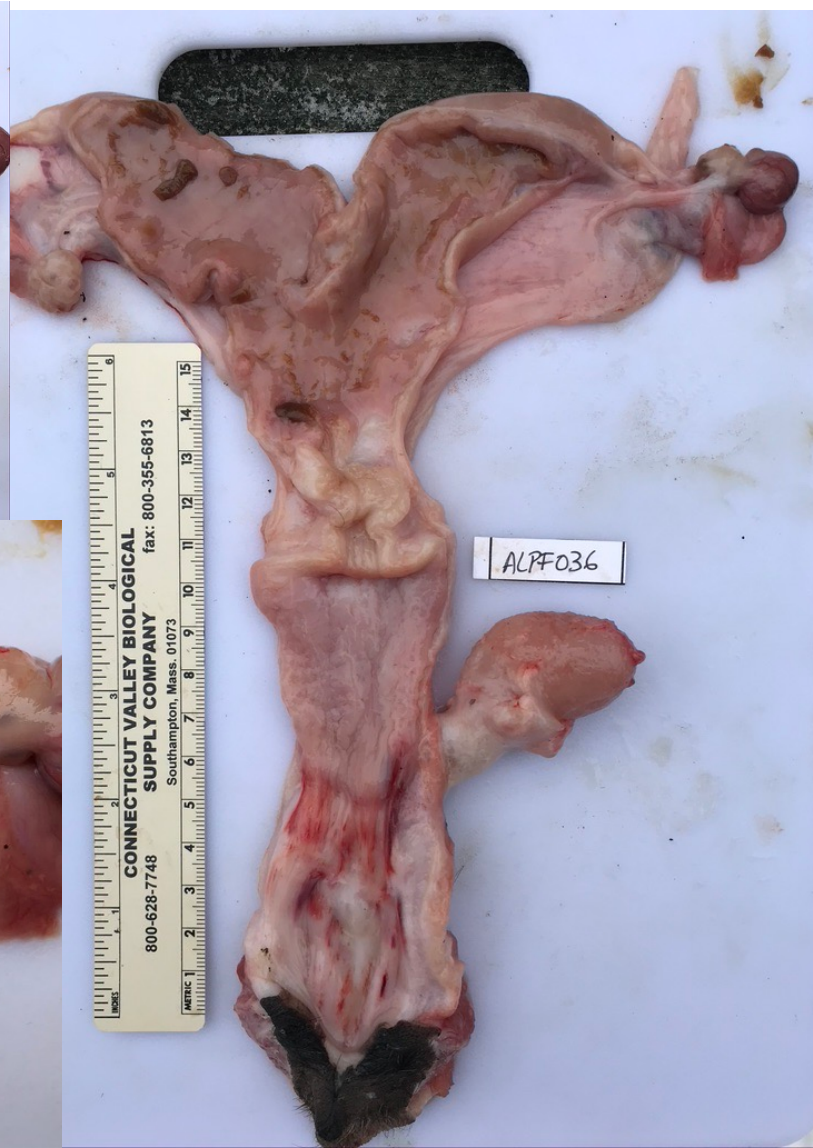

ALP037

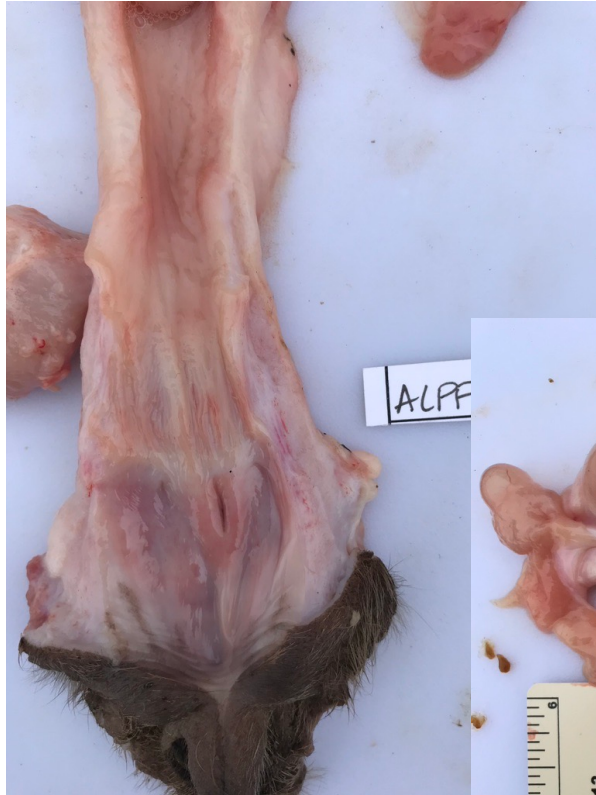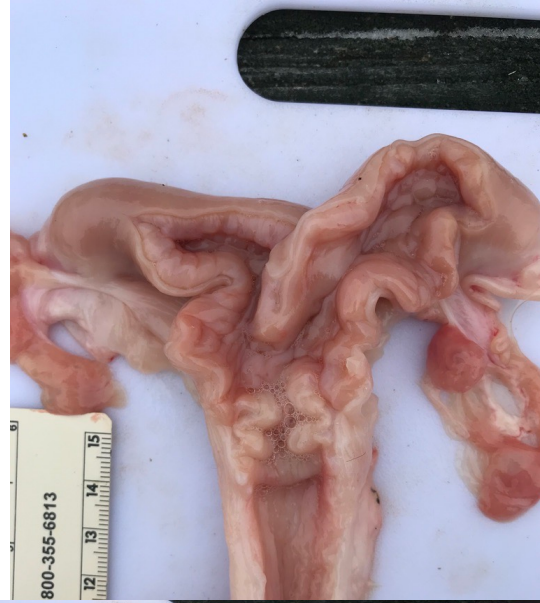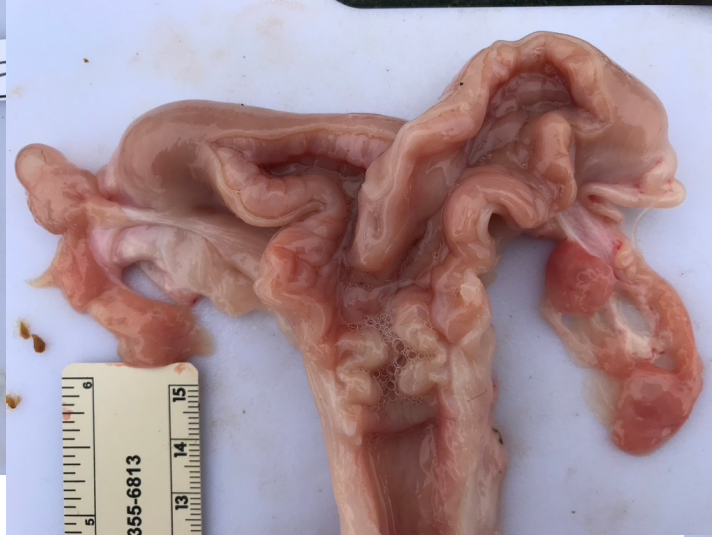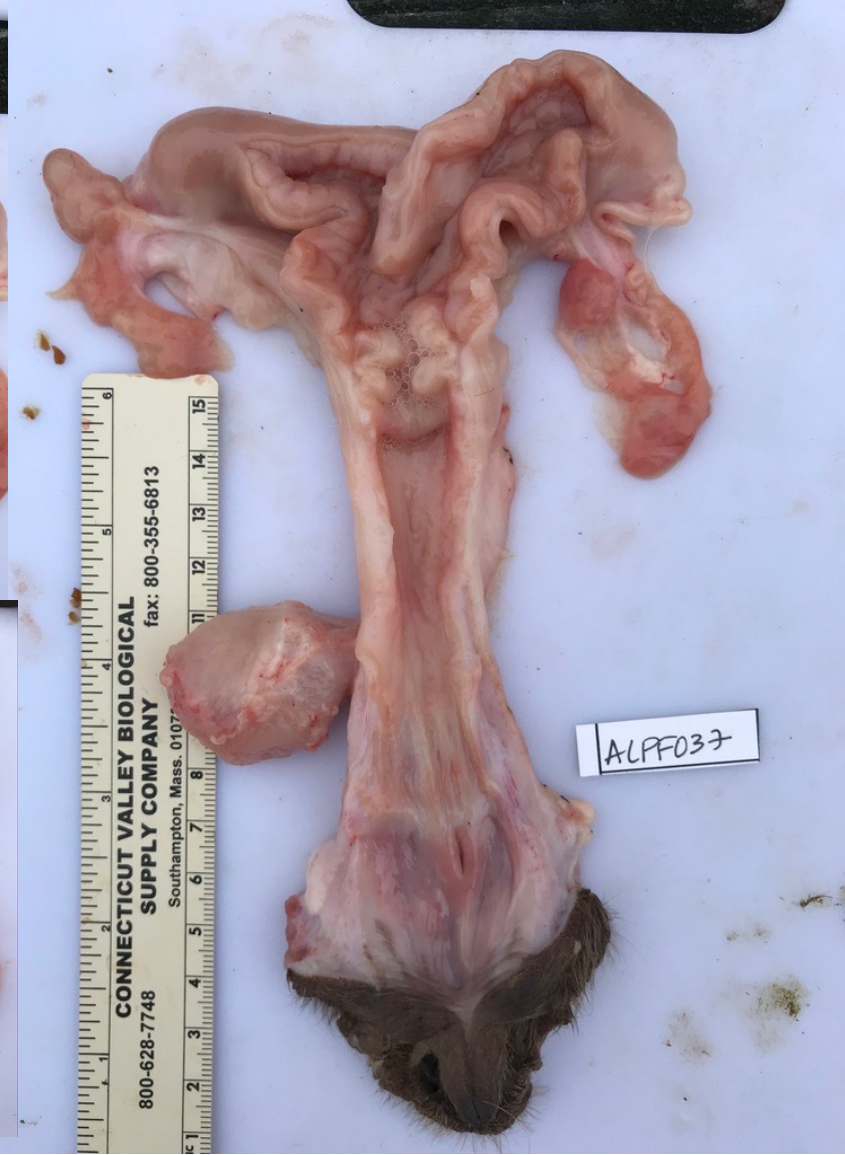

ALP039

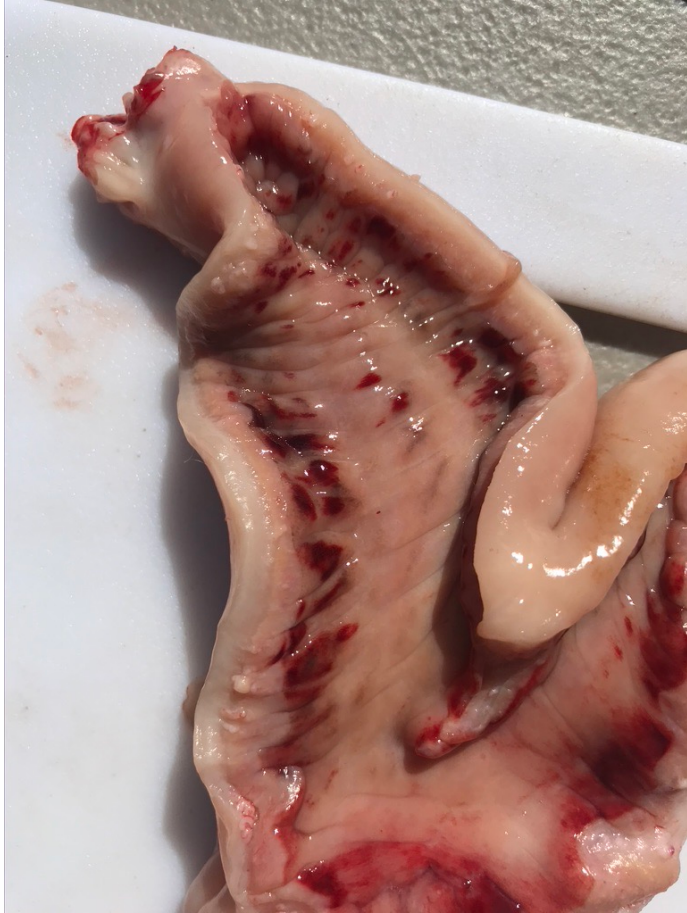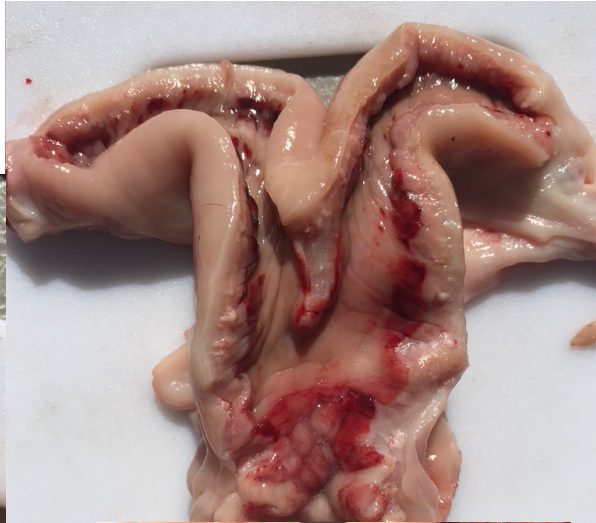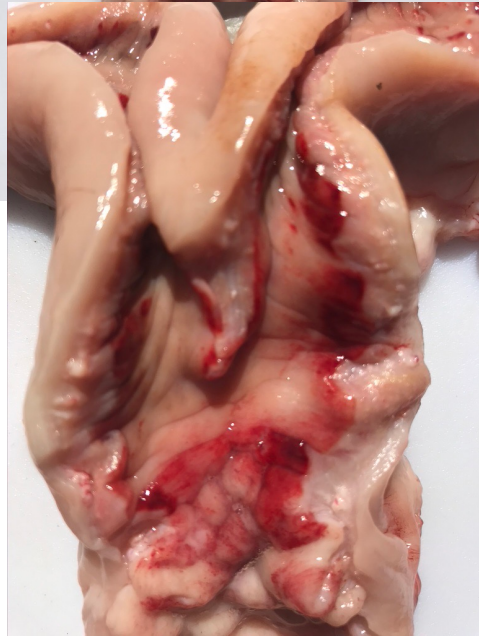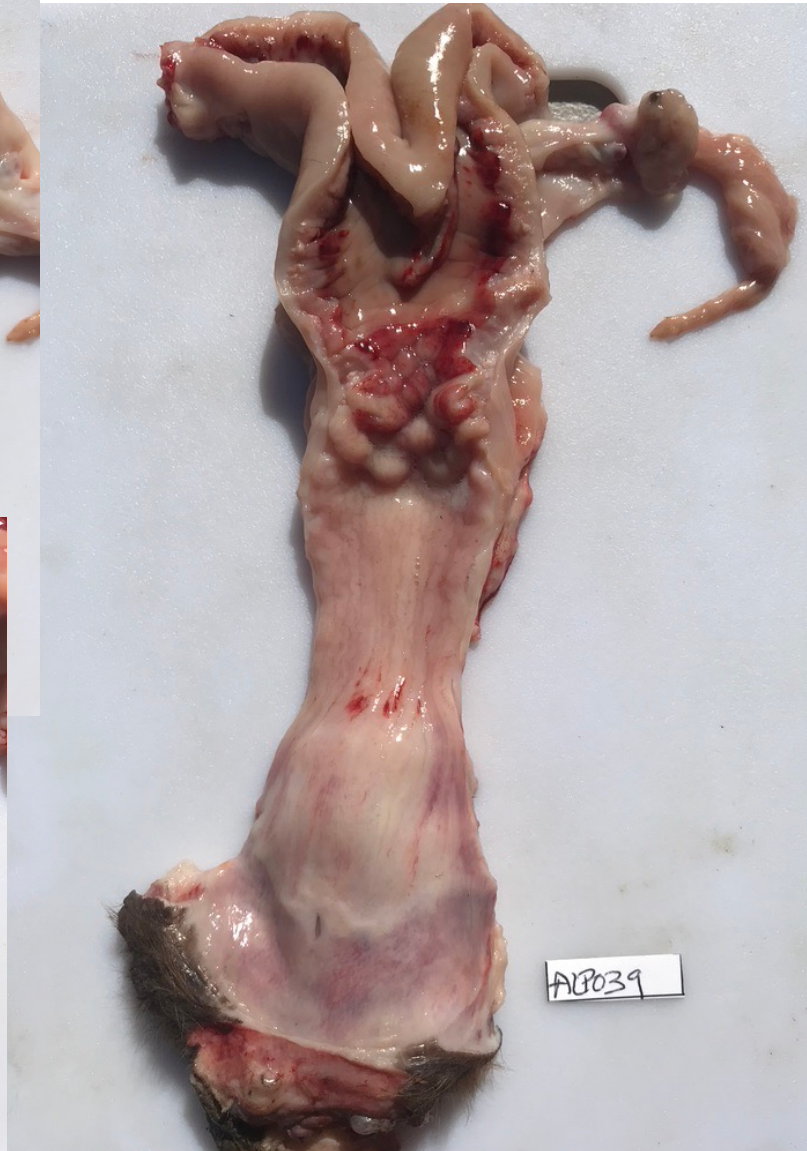

ALP040

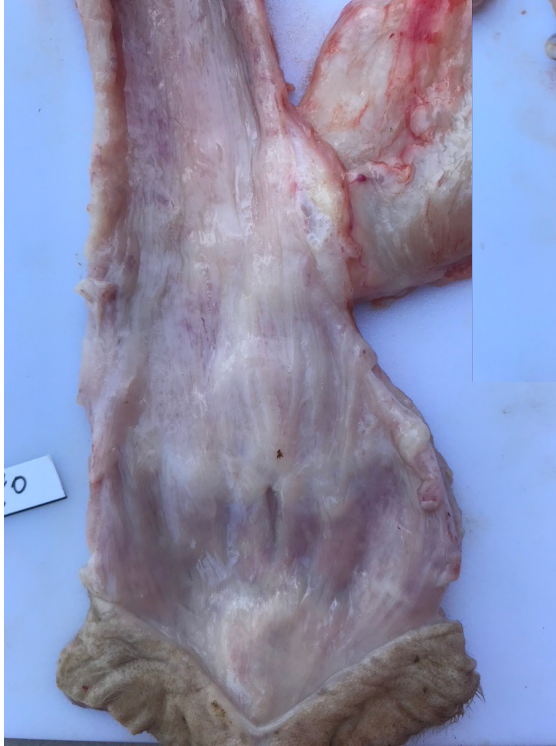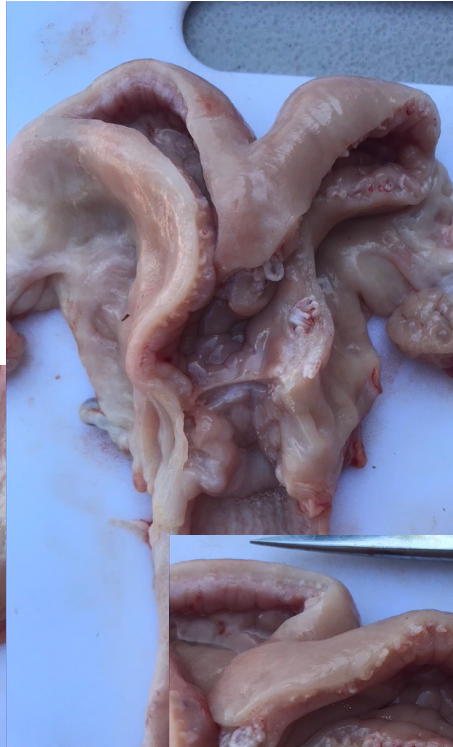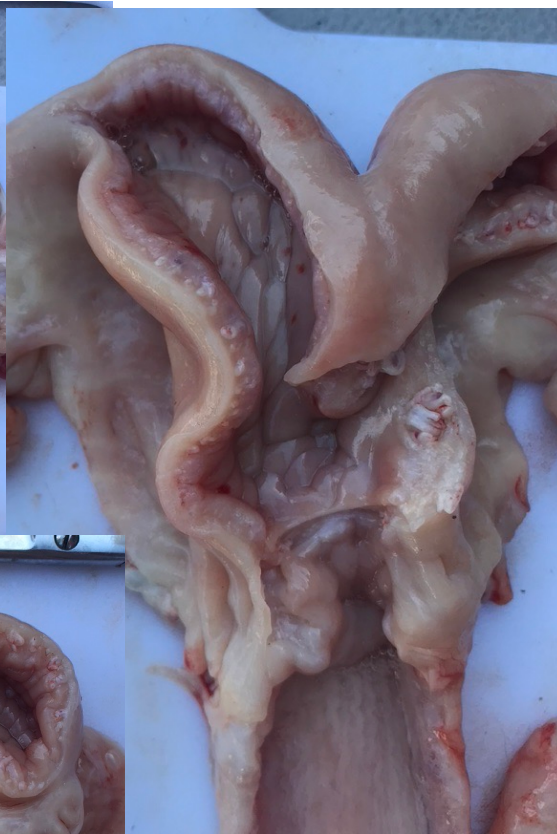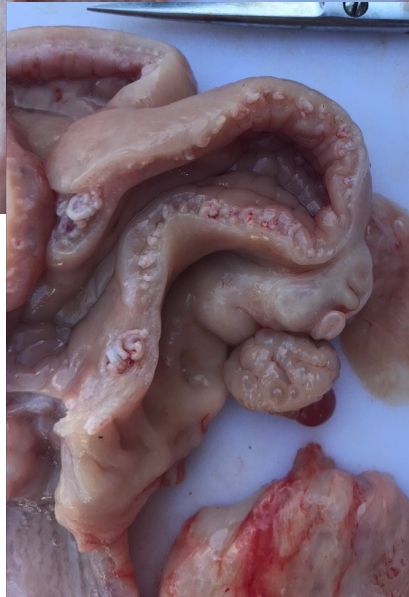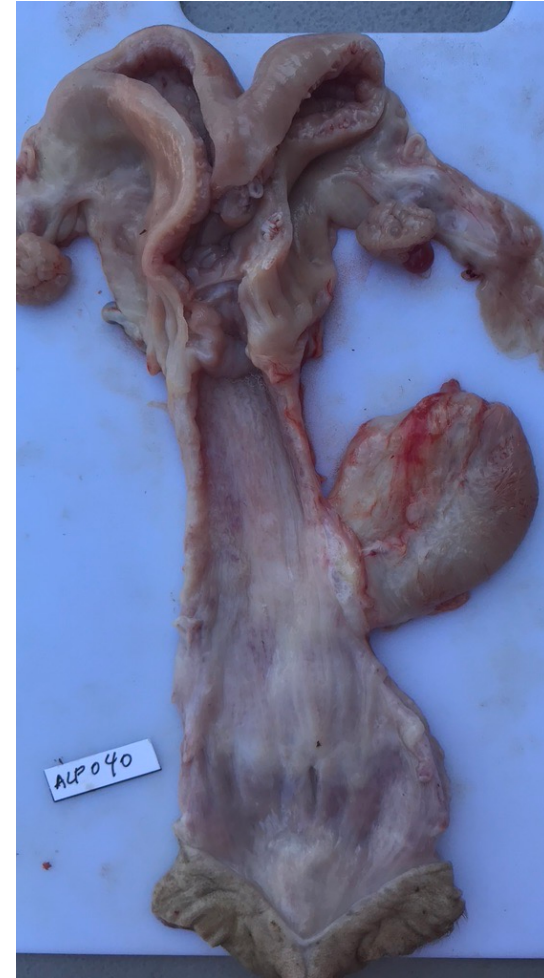

ALP041

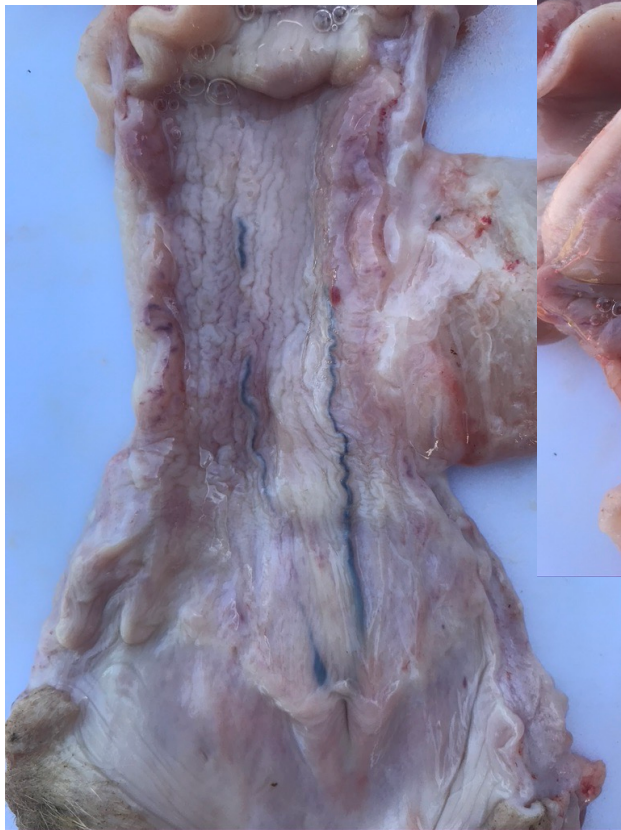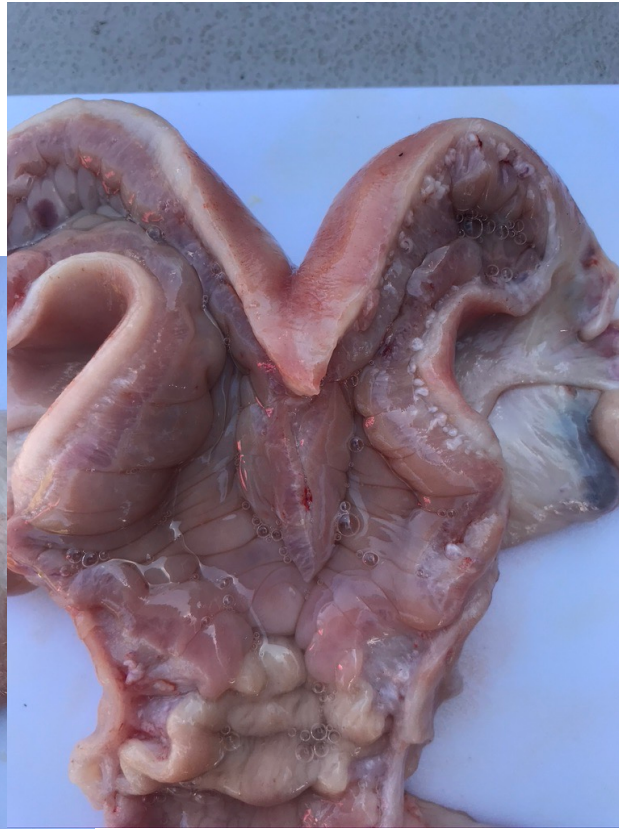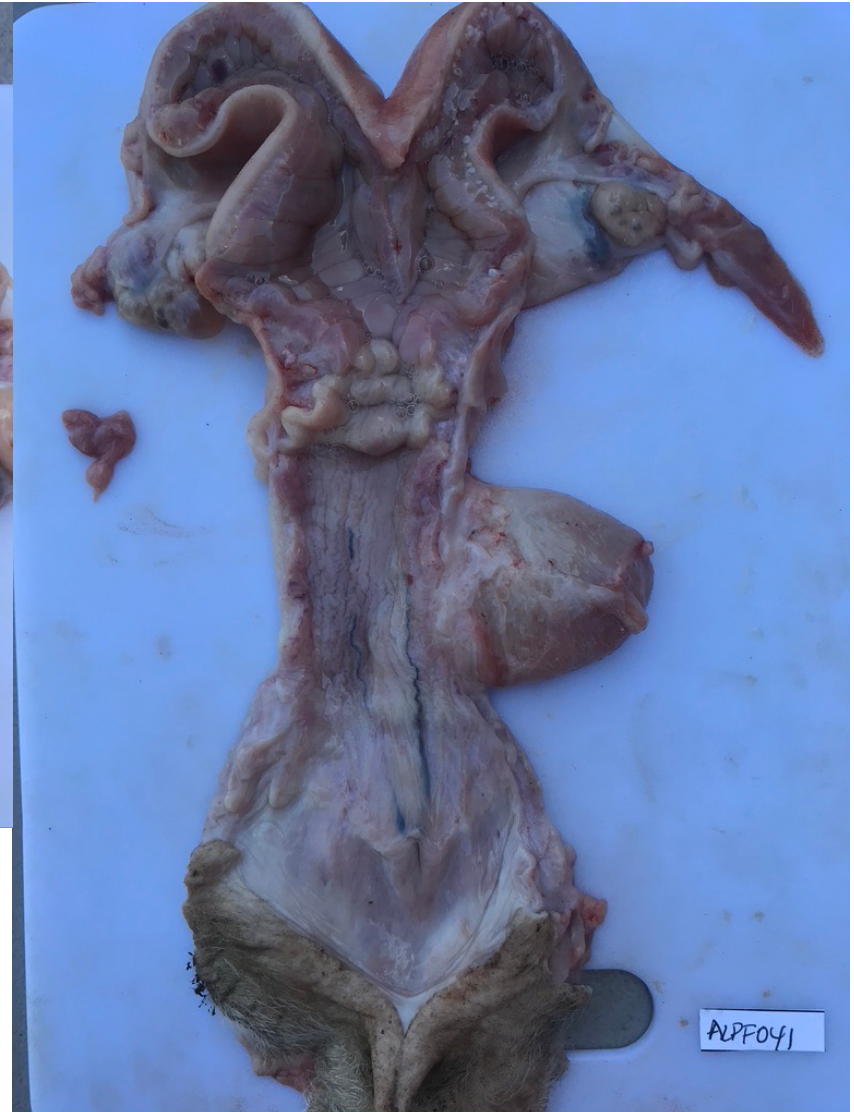

ALP042

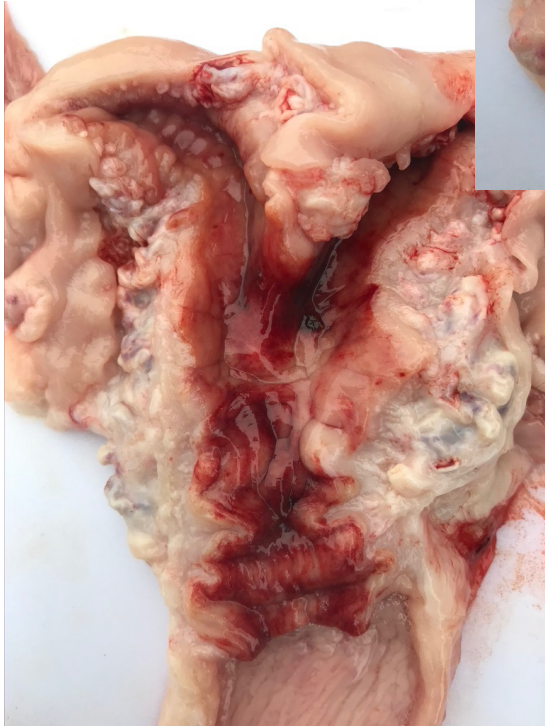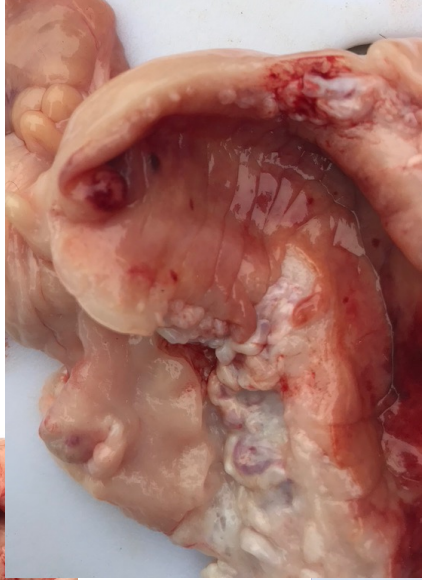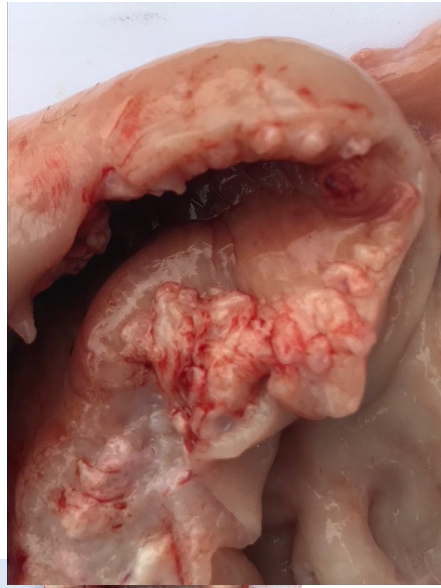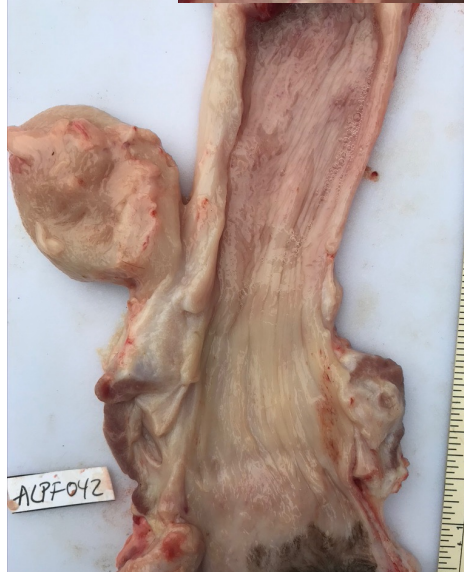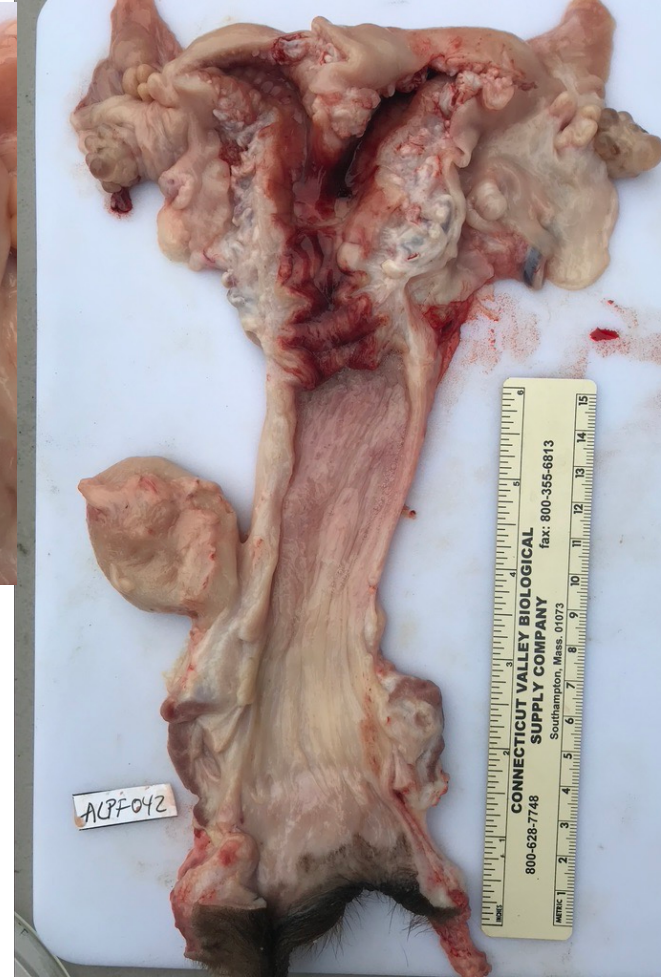

ALP043

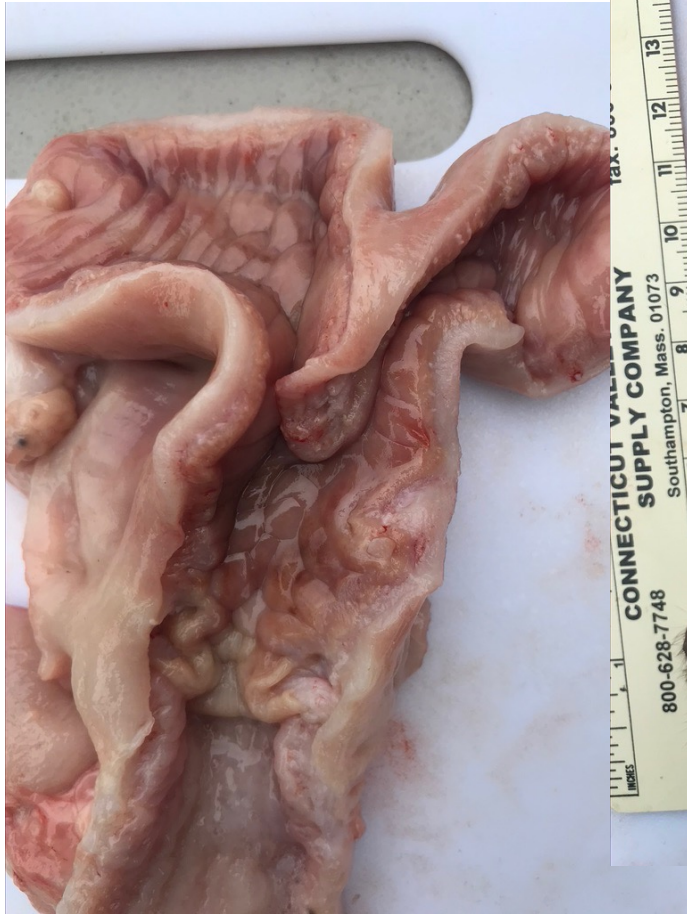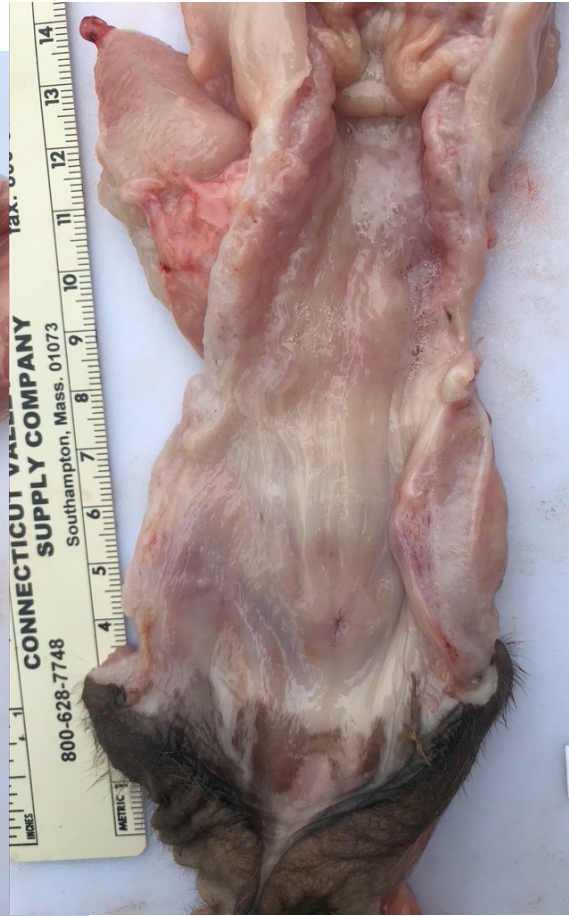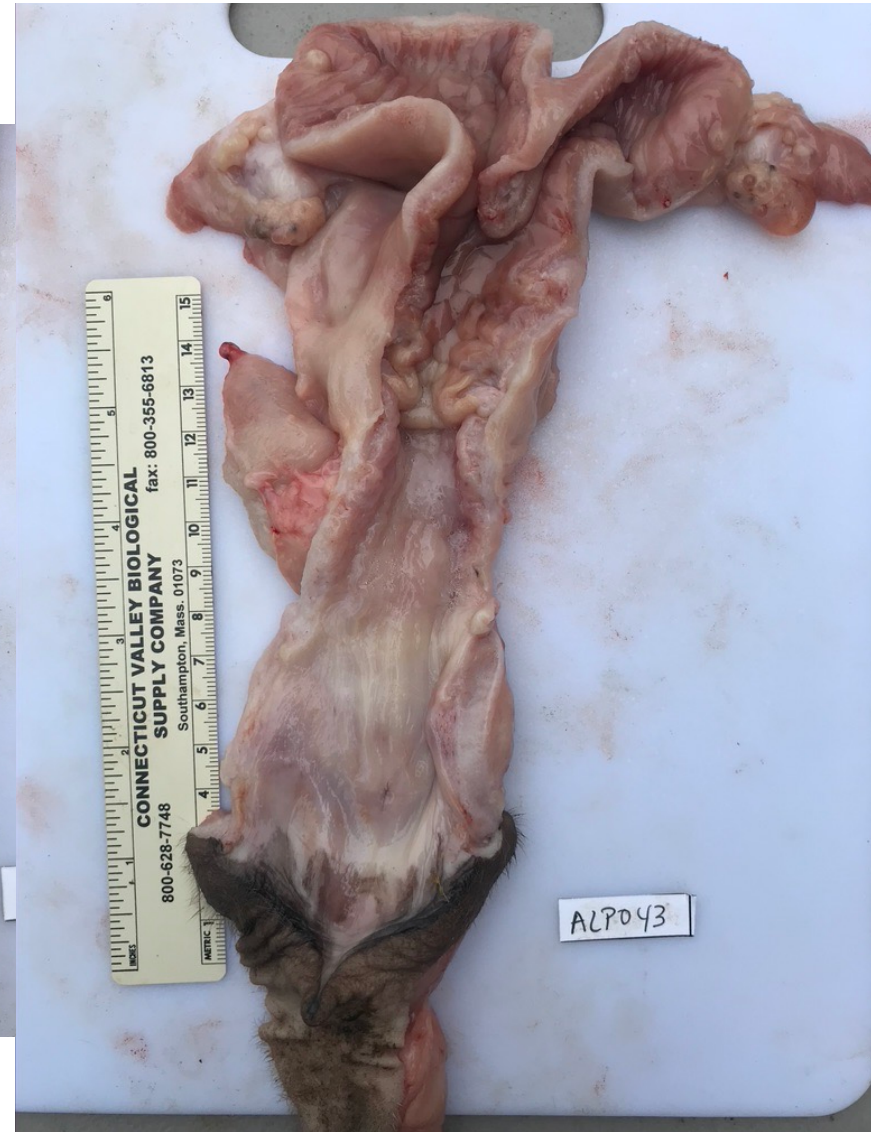

ALP044

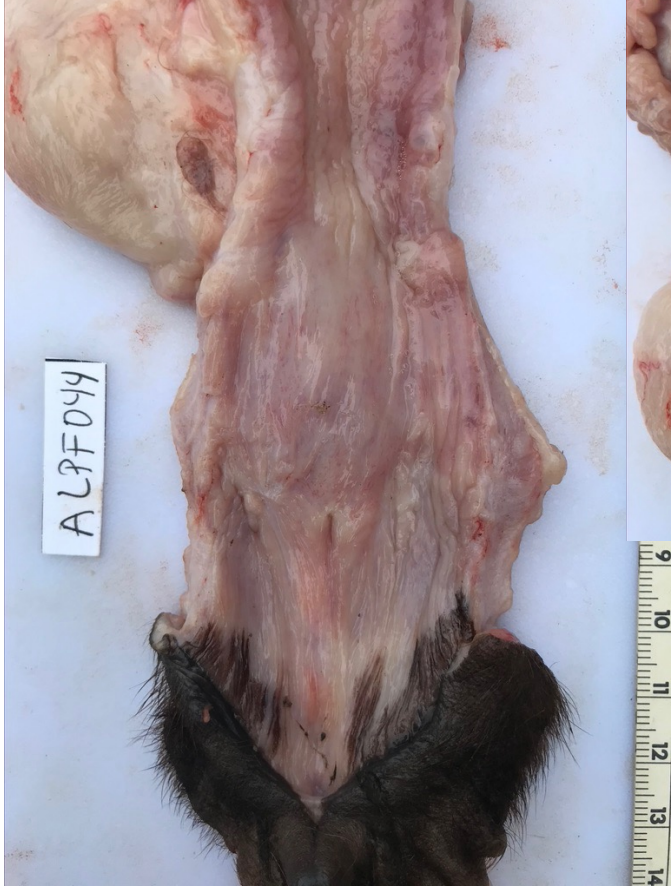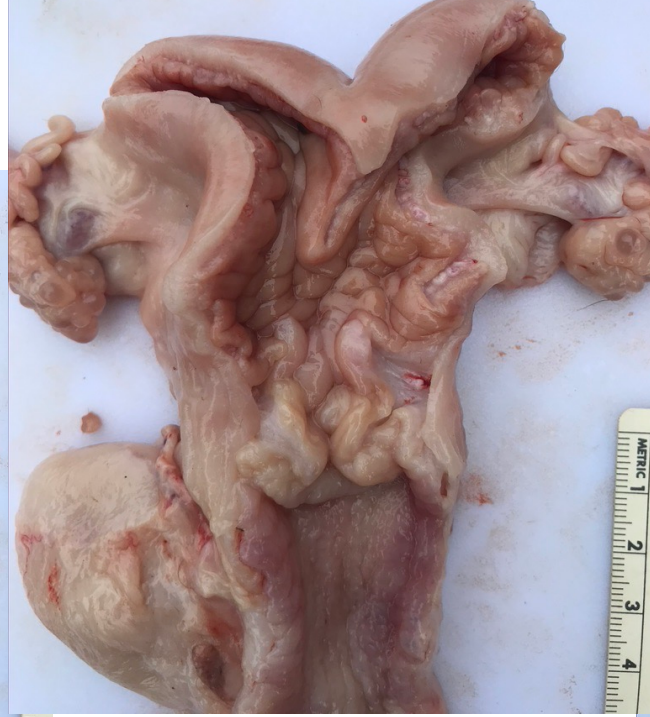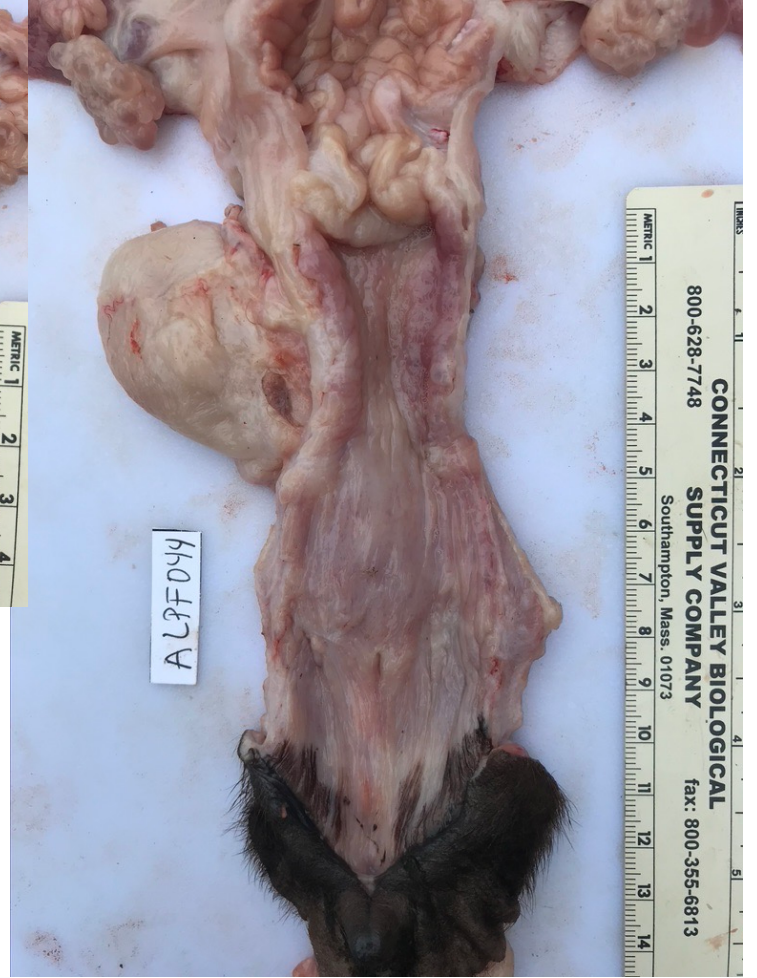

Supplement: S1 Fig — (PDF) [file pone.0295882.s004.pdf]
