# Supplementary material for: Intra-horn insemination in the alpaca Vicugna pacos: Copulatory wounding and deep sperm deposition
Source: PLoS One. 2024 Apr 17;19(4):e0295882. doi: 10.1371/journal.pone.0295882 (PMC11023217; doi:10.1371/journal.pone.0295882)
Supplement: Attachment — Submitted filename: Response to reviewers Brennan et al.docx [file pone.0295882.s006.docx]

Response to reviewers

Brennan, Purdy and Bacon

Re: Resubmission of MS PONE-D-23-40086

The reviewers had very minimal comments and we have addressed all of them below.

*R1: Given the wide audience of Plos one, the introduction would greatly benefit from a diagram of the alpaca reproductive system.*

AU: We already have a labeled photo in Fig 1, therefore we did not add a diagram.

*R1: Local farm where?*

AU: Connecticut. Added

*R1: Examined and photographed immediately after euthanized?*

AU: Yes, added

*R1: It may help other researchers to know what problems were faced with sample preservation of slides, did the stain not work? That is important information so other researchers don’t make the same mistake.*

AU: We realized that these slides were missing because on the females mated 1hr before euthanasia we had not started yet to use the diff quick stain that allowed us to see immune cells but were using eosin nigrosin stain for live/dead sperm. We have deleted the sentence and instead rewrote this paragraph in the manuscript as follows: “We then used a glass slide to touch each region of the inside of the reproductive tract and stained these slides either with a modified Giemsa stain (Differential Quik Stain Kit, VWR, Radnor PA) to look for the presence of inflammatory cells (unmated and mated +24 hrs) and sperm (mated +24 hrs), or with Semen Morphology Stain (eosin/nigrosin) (mated +1hr)”.

*R1: Three values after the 0 for p values is more than enough.*

AU: Fixed

*R1: I suggest values for Table 1 should be a graph, with averages or percentages, making data easier to visualize.*

AU: Added Figure 2. Changed the figure captions for all remaining figures

*R1: Discussion, “female cooperation and receptivity are required for copulation to succeed because copulation is prolonged”not quite following the argument as to why female cooperation is necessary because copulation is long.. In insects copulations can be very lengthy, much more than the 20 min listed here for alpacas, but there still can be sexual conflict if the male is coercing or manipulating the female into mating with him… So I would suggest perhaps limiting this discussion to the ways in which females need to adapt certain positions to allow intromission. Also, is there any evidence for females controlling copula duration as in tephritid flies?*

AU: We modified this paragraph, now it reads as follows: “However, it seems unlikely that alpacas experience the usual selective pressures that may induce copulatory wounding as a result of sexual conflict; females typically do not mate with multiple males because a single dominant male controls reproductive access to the female group, and female cooperation and receptivity are required for copulation to succeed because females must adopt a prone position to allow intromission, and the lengthy copulation would allow females plenty of opportunity to reject the male by changing position. Either males or females may terminate the copulation, but usually the male stops anywhere between 5-60 minutes after intromission, by standing up. Other times the female may push up or roll to her side and terminate copulation”.

Reviewer #2: *This is an interesting study of the alpaca natural insemination, there are some mistakes and clarifications and I included my comments in the article PDF attached with this format, for this publication, it is important to know how the animals were cared for this study, the ethic component*.

Reviewer 2 had 8 spelling and formatting corrections and we have fixed all of them. Since R1 also had a question about housing of the alpaca we added the following statement: “We obtained whole reproductive tracts from adult female alpacas that were being culled for meat processing at a farm in Connecticut, USA, where animals are maintained in accordance with State and federal guidelines concerning livestock management in small farms”
